# Unlocking COVID therapeutic targets: A structure-based rationale against SARS-CoV-2, SARS-CoV and MERS-CoV Spike

**DOI:** 10.1016/j.csbj.2020.07.017

**Published:** 2020-07-31

**Authors:** João Trigueiro-Louro, Vanessa Correia, Inês Figueiredo-Nunes, Marta Gíria, Helena Rebelo-de-Andrade

**Affiliations:** a Antiviral Resistance Lab, Research & Development Unit, Infectious Diseases Department, Instituto Nacional de Saúde Doutor Ricardo Jorge, IP, Av. Padre Cruz, 1649-016 Lisbon, Portugal; b Host-Pathogen Interaction Unit, Research Institute for Medicines (iMed.ULisboa), Faculty of Pharmacy, Universidade de Lisboa, Av. Professor Gama Pinto, 1649-003 Lisbon, Portugal

**Keywords:** aa, amino acid, ACE2, angiotensin-converting enzyme2, Bat-SL-CoVs, bat SARS-like coronavirus, Beta-CoVs, betacoronavirus, CC, conserved cluster, CD, connector domain, CDR, consensus druggable residue, CDP, consensus druggable pocket, CH, central helix, CoVs, coronavirus, CP, cytoplasmic domain, CR, connecting region, CS, conservation score, DGSS, DoGSiteScorer, DPP4, dipeptidyl peptidase-4, FP, fusion peptide, HR1, heptad repeat 1, HR2, heptad repeat 2, hSARSr-CoVs, human Severe acute respiratory syndrome-related coronavirus, MERS-CoVs, middle east respiratory syndrome coronavirus, MERSr-CoVs, middle east respiratory syndrome-related coronavirus, MSA, multiple sequence alignment, NTD, N-terminal domain, nts, nucleotides, PDB, Protein Data Bank, PDS, PockDrug-Server, RBD, Receptor-Binding Domain, S, Spike, SARS-CoVs, severe acute respiratory syndrome coronavirus, SARS-CoV-2, severe acute respiratory syndrome coronavirus 2, SARSr-CoVs, severe acute respiratory syndrome-related coronavirus, SD1, subdomain 1, SD2, subdomain 2, Sv, shorter variant, SF, SiteFinder from MOE, SP, small pocket, TMPRSS2, transmembrane protease serine 2, T-RHS, top-ranked hot spots, Betacoronavirus, Coronavirus disease, Druggability prediction, Novel antiviral targets, SARS-CoV-2, Sequence conservation, Spike protein

## Abstract

There are no approved target therapeutics against SARS-CoV-2 or other beta-CoVs. The beta-CoV Spike protein is a promising target considering the critical role in viral infection and pathogenesis and its surface exposed features. We performed a structure-based strategy targeting highly conserved druggable regions resulting from a comprehensive large-scale sequence analysis and structural characterization of Spike domains across SARSr- and MERSr-CoVs. We have disclosed 28 main consensus druggable pockets within the Spike. The RBD and SD1 (S1 subunit); and the CR, HR1 and CH (S2 subunit) represent the most promising conserved druggable regions. Additionally, we have identified 181 new potential hot spot residues for the hSARSr-CoVs and 72 new hot spot residues for the SARSr- and MERSr-CoVs, which have not been described before in the literature. These sites/residues exhibit advantageous structural features for targeted molecular and pharmacological modulation. This study establishes the Spike as a promising anti-CoV target using an approach with a potential higher resilience to resistance development and directed to a broad spectrum of Beta-CoVs, including the new SARS-CoV-2 responsible for COVID-19. This research also provides a structure-based rationale for the design and discovery of chemical inhibitors, antibodies or other therapeutic modalities successfully targeting the Beta-CoV Spike protein.

## Highlights

• Human SARSr-CoV Spike protein is highly conserved in both S1 and S2 subunits.
• The S1-RBD, -SD1; S2-CR, -HR1 and -CH are the most promising druggable S regions.
• 28 main consensus druggable pockets were found with high druggabbility score.
• New hot spots were identified for hSARSr-CoVs (n = 181) and Beta-CoVs (n = 72).
• A structure-based rationale is disclosed for drug design and discovery.

## Graphical abstract

**Figure:**
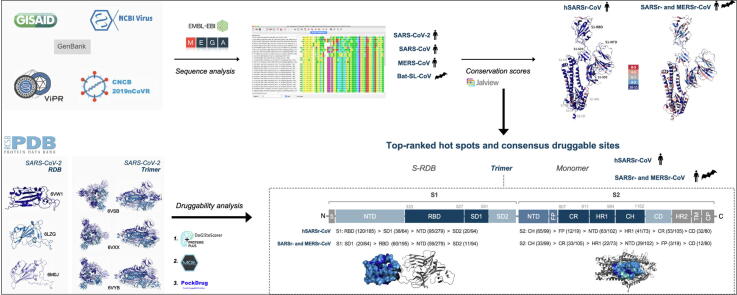

## 1 Introduction

There are no antiviral drugs approved specifically for the prevention or treatment of coronavirus disease 2019 (COVID-19) caused by SARS-CoV-2; or against any other coronavirus (CoVs).

The current recommendations for the clinical management of COVID-19 include infection prevention and control measures and supportive care [1]. Because there is no sufficient information to recommend for or against broad spectrum antivirals, repurposing drugs or immunomodulatory therapy, moderate to critically ill patients have been managed with these alternative therapies through Emergency Use Authorizations, Emergency Investigational New Drug applications, compassionate use or expanded access programs with drug manufacturers [1], [2].

The molecular epidemiology of SARS-CoV-2 in the post-pandemic period is uncertain. It may remain endemic or follow a re-emerging epidemiology dynamics which, in addition to the continuous public health threat of zoonotic CoVs, makes the development of new antiviral drugs crucial. Among the most promising viral targets is the Spike (S) protein, which has a critical role in viral infection and pathogenesis. It is surface exposed and mediates the entry of CoVs in the host cell, which makes it the main target of neutralizing antibodies and an obvious antiviral target for new drug development [3], [4], [5], [6], [7].

Two main features have been associated with the zoonotic potential of CoVs and are important determinants of infectivity, pathogenesis and host range, the receptor recognition and a multi-basic cleavage S1/S2 site for priming of the S protein conferring high-cleavability [3], [8], [9], [10]. Receptor binding induces conformational changes in the S protein, exposing cleavage sites for priming proteases [11]. Binding to a receptor is mediated by a receptor-binding domain (RBD), which is located in the surface unit S1. The S1 subunit generally consists of an N-terminal domain (NTD) and a C-terminal domain, which can serve as RBD either alone or in combination [11]. For SARS-CoV-2 S protein, Angiotensin Converting Enzyme-2 (ACE2) has been identified as the primary receptor, as for SARS-CoVs who also belongs to the B lineage of betacoronavirus (beta-CoVs), but not for MERS-CoVs from the C lineage which uses human dipeptidyl peptidase 4 (DPP4) [3], [4], [11], [12], [13], [14].

Priming occurs at S1/S2 interface and S2′sites, providing the CoV S protein with the structural flexibility required for the membrane fusion reaction. The cleavage at S2́ generates the mature N-terminus of the fusion peptide (FP), that is inserted into the membrane [11]. In SARS-CoV-2, SARS-CoVs and MERS-CoVs the priming proteolytic cleavage process is carried out by human Transmembrane Protease Serine 2 (TMPRSS2), although MERS-CoVs require a pre-cleavage for subsequent S protein activation by TMPRSS2 carried out by furin in infected cells [4], [11], [15], [16], [17], [18], [19].

The aim of this study was to identify and map druggable consensus hot spots or regions in the three-dimensional structure of the S protein of SARS-CoV-2, that can act as antiviral targets for the development of new molecules against a broad-range of beta-CoVs. This research also contributes with a new panel for Spike structure–function studies, which can accelerate the Spike target validation and prompt in silico-chemico-biological approaches that aid in the discovery of potent antiviral drugs or monoclonal antibodies. We used a comprehensive approach, combining data from a conservation analysis of amino acid (aa) sequences from Beta-CoVs (lineages B and C), with data from a druggability study on SARs-CoV-2 crystallographic structures.

Anti-coronavirus strategies based on highly conserved and druggable targets are lacking. Structure-based design approaches have proven fundamental in the discovery and development of new drugs, including influenza antivirals; and can reduce the time and costs associated to the *de novo* drug development [20], [21]. Also, predicting the druggability of the targets is a critical step in drug discovery, particularly since undruggable targets are responsible for up to 60% of failure in this process [22], [23].

## 2 Results and discussion

### 2.1 Spike length and underlying variations in primary protein structure

S length varies across all four beta-CoVs. SARS-CoV-2 harbours a protein 18-aa longer than the SARS-CoVs (1273 aa); and a high inter-variability in length was observed in bat-SL-CoV S sequences (1128 to 1269 aa), concordant with the high diversity of bat-SL-CoVs found in bat populations (Table 1) [24]. MERS-CoVs harbour the longest S protein, with further 80 aa than the SARS-CoV-2 (1353 aa; Table 1). The differences in S length are entirely attributed to differences in the S1 subunit, except in the case of MERS-CoVs (Table 1). Fifteen different length variation types were found in bat-SL-CoV S1 sequences (640–681 aa range), with bat/Yunnan/RaTG13/2013 (RaTG13) and LYRa11/R.affinis/Yunnan/2011 (LYRa11) viruses harbouring the S1 subunit most similar in length to SARS-CoV-2 S1 (681 and 671 aa, respectively). RaTG13 clusters with SARS-CoV-2 in a distinct evolutionary group from other severe acute respiratory syndrome-related coronavirus (SARSr-CoVs) [5], whereas LYRa11 is a potential recombinant descended from parental lineages considered as a gap-filling virus between bat-SL-CoVs and human SARS-CoVs [25]. Overall, 5 deletions and 10 insertions of variable length distinguished bat-SL-CoV from SARS-CoV-2 S1 aa sequences. Minor differences were found between SARS-CoV and SARS-CoV-2 S1 primary structure, namely a single 4-aa insertion and 6 deletions (3–7 aa), while MERS-CoV S1 aa sequence differed from the novel virus in 19 insertions (1–13 aa) and 13 short deletions (1–4 aa). Most of the residue insertions (1/1 SARS-CoVs, 3/5 bat-SL-CoVs, and 11/19 MERS-CoVs) and most (4/6 SARS-CoVs) or half (5/10 bat-SL-CoVs, 6/13 MERS-CoVs) of the aa deletions observed take place in the S1-NTD, evidencing that this specific domain might not be a suitable target for a wide-reaching antiviral strategy against all four beta-CoVs. S1-NTD is responsible for binding sugar receptors for cell entry and differences in this domain may result in an altered specificity for a different sugar receptor [3]. In fact, NTD of middle east respiratory syndrome-related coronavirus (MERSr-CoVs) binds preferably to α2,3-linked sialic acid, and no sugar binding has been reported for SARS-CoVsNTD [13]. Whether or not SARS-CoV-2 NTD binds to sugar remains unknown to date. At the S1/S2 boundary there is a polybasic cleavage site, between aa 682 to 685 (SARS-CoV-2 numbering), in SARS-CoV-2 (RRAR) and MERS-CoVs (RSVR) (Table 1), but not in SARS-CoVs and bat-SL-CoVs, in which the S protein remains uncleaved during assembly and exocytosis. The presence of a S1/S2 cleavage site in MERS-CoVs revealed to be important, but not essential, for S protein activation [26], [27]. Whether or not this extends to SARS-CoV-2 S protein remains to be determined, leaving the path open for a potential virus-specific antiviral strategy.

**Table 1:** Distinctive features (length variation and cleavage site) and number of sequences enclosed in the datasets of the Spike S1 and S2 subunits of each betacoronavirus analysed.

Genus	Subgenus	Species	Lineage	Virus	**SPIKE PROTEIN** (S gene)											
					**Whole-protein**			**S1 subunit**				**S2 subunit**				Polybasic **S1/S2** cleavage site ^d^
					Position in the genome (nts) ^a,b^	Lenght		Position in the genome (nts) ^a^	Lenght		Number of sequences	Position in the genome (nts) ^b^	Lenght		Number of sequences	
						nts	aa		nts	aa			nts	aa		
**Beta-CoV**	Sarbecovirus	SARSr-Cov	B	SARS-CoV-2	21314–25135	3822	1273	21314–23368	2055	685	674	23369–25135	1767	588	682	^682^**RRAR**^685^
				SARS-CoV	21228–24995	3768	1255	21228–23228	2001	667	114	23229–24995	1767	588	116	–
				bat-SL-CoV	21,264 - [24950–25073]	3687–3810	1228–1269	21,264 - [23183–23306]	1920–2043	640–681^c^	50	[23184–23307] - [24950–25073]	1767	588	50	–
	Merbecovirus	MERSr-Cov	C	MERS-CoV	21178–25239	4062	1353	21178–23430	2253	751	248	23431–25239	1809	602	248	^682^**RSVR**^685^
										TOTAL	**1086**			TOTAL	**1096**	

^a^ counting from the start codon of the first genome-encoded protein (nsp1); ^b^ includes the STOP codon; ^c^ 15 different lengths: 649 (15/50); 648 (9/50); 653 (7/50); 663 (5/50); 640, 668, and 667 (2/50); 681, 662, 657, 658, 647, 654, 646, and 671 (1/50); ^d^ SARS-CoV-2 numbering

The S2 subunit of SARSr-CoVs are equal in length (588 aa); whereas the MERS-CoVsS2 is 14-aa longer (602 aa; Table 1), having been identified 9 insertions (1–5 aa) and 3 short deletions (1–4 aa), compared to SARSr-CoVsaa sequence. Four of the 9 insertions lie at the central helix (CH), which may enable a slightly different conformation of the helix promoted by the binding to a different receptor [5].

### 2.2 Conservation studies

#### 2.2.1 SARS-CoV-2

The S protein of the SARS-CoV-2 is highly conserved. In the S1 and S2 subunits, 99.8% and 95.6% of the residues are highly conserved, respectively (conservation score (cs) ≥ 10). In S1-SD2, only position 614 was found to be variable (68.7% D614 vs 31.3% G614). There is a widespread geographic distribution of the D614G mutation since SARS-CoV-2 strains bearing this mutation were found in all continents. The majority of strains isolated in North and South America, Oceania and Asia, contain a D residue at position 614, while most of strains from Europe, Africa and Central America contain the substitution D614G. Conversely, in the S2 subunit, 10 variable residues (cs < 7) were found at positions D839, A852, V860, L861 (connecting region (CR) between FP and heptad repeat 1 (HR1) domains); S939, S940 and S943 (in HR1); V1040 (in CH); and C1254 and P1263 (in cytoplasmic domain (CP)). Specifically, the variants D839Y and D839E can be identified; the variant A852V occurs in strains from Netherlands; V860Q and L861K co-appear in the same strain from Beijing; S939F occurs exclusively in strains from Europe; S940F and C1254 occur in Australia isolates; S943P and S943T variants are found in Belgium isolates; V140F appears in strains from China; and P1263L occurs in strains from England and Henan. The supplemental Figure S-1a displays the SARS-CoV-2 conservation-score distribution for the S S1 and S2 subunits.

The S-RBD, described in the literature as a promising antiviral target [28], [29], [30], contains conserved and highly conserved residues, presenting only minor aa variations with equivalent aa properties: five strains isolated in France present V367F and four strains isolated in the USA bear the aa substitution V483A.

#### 2.2.2 SARSr- and MERSr-CoVs

The S protein is mostly conserved among SARSr-CoVs (SARS-CoV-2, SARS-CoVs and bat-SL-CoVs). In regard to human SARSr-CoVs (hSARSr-CoVs), 64.7% of the residues within the S1 subunit are highly conserved (cs ≥ 10); 11.2% are conserved (cs of 7–9) and only 24.1% are considered variable (cs ≤ 6.9). Most variable sites/residues are found in S1-NTD and at the C-terminal end of SD2. The S2 subunit is mostly highly conserved: 90% of the residues are highly conserved, 2.5% are conserved and only 7.2% are variable (mostly located in the S2-NTD and in the CR. A similar conservation pattern was found in bat-SL-CoVs.

In regard to SARSr- and MERSr-CoVs (SARS-CoV-2, SARS-CoVs, bat-SL-CoVs and MERS-CoVs), in the S1 subunit 20.2% of the residues are highly conserved, 36.7% are conserved, and 43.1% are variable. In the S2 subunit, 42.2% of the residues are highly conserved, 23.8% are conserved and 34% are variable.

Variable sites/residues are found in SARS-CoV-2, SARSr-CoVs, and between SARSr- and MERSr-CoVs. It possibly suggests strong selective pressure; and, in this context, these residues or regions do not constitute, *a priori*, suitable regions for the design of a promising antiviral strategy targeting the S protein. The Figure S-1 displays the S conservation-score distribution for the hSARSr-CoVs (Figure S-1b) and for the SARSr- and MERSr-CoVs (Figure S-1c).

**Fig. 1:**
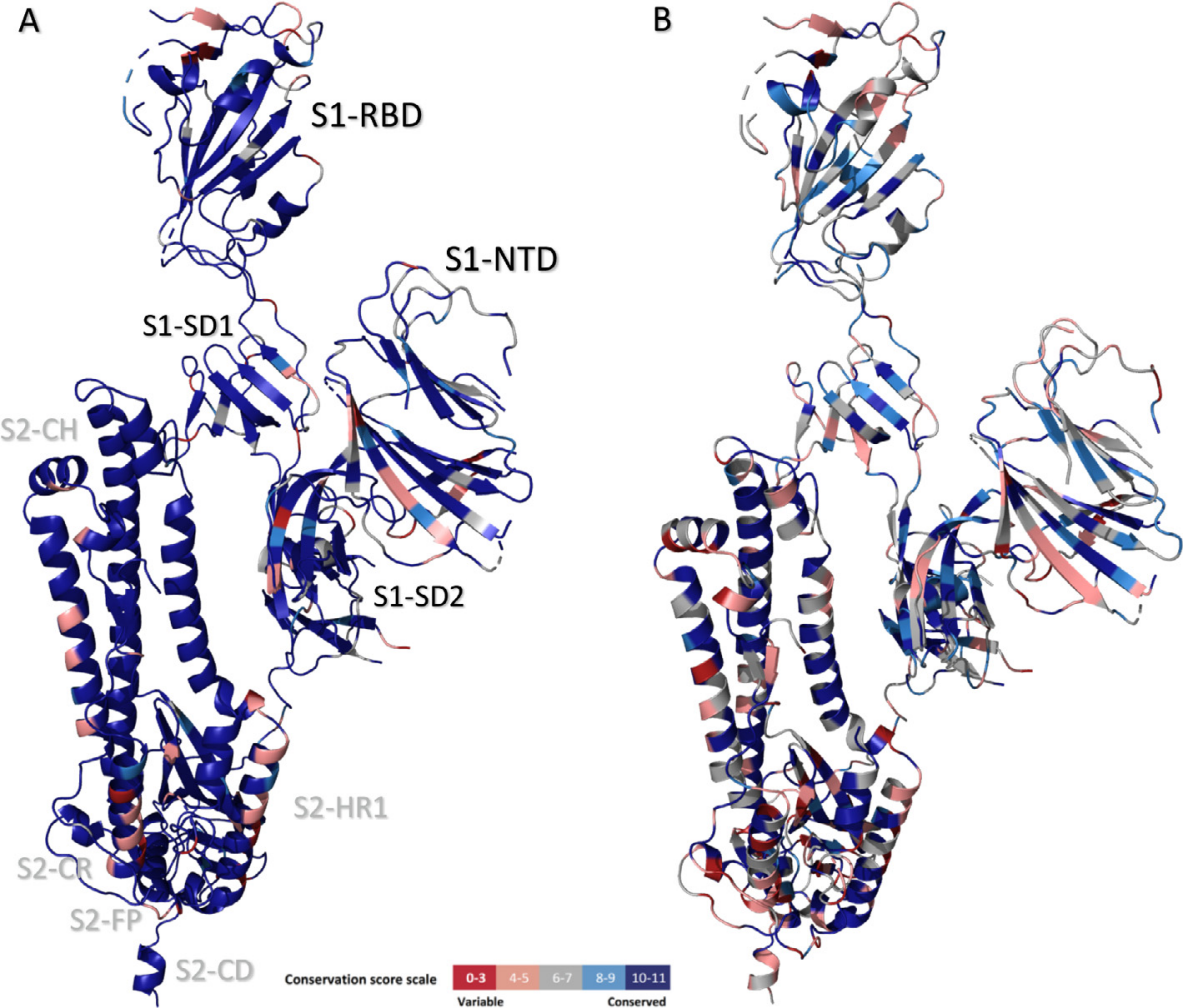
**Three-dimensional sequence conservation of the human SARSr-CoVs (A) and SARSr- and MERSr-CoVs (B) Spike protomers (PDB ID: 6VYB**). The cs are mapped onto the S protein structures. The figure is coloured based on cs: the highest conservation positions are highlighted in shades of blue and the red regions indicate low conservation according to the cs scale. The S1 and S2 domains resolved are indicated in black and grey, respectively, in the hSARSr-CoV protomer. NTD: N-terminal domain; RBD: receptor-binding domain; SD: subdomain; FP: fusion peptide; CR: connecting region, HR: heptad repeat; CH: central helix. The figures were produced with PyMOL Molecular Graphics System [101]. (For interpretation of the references to colour in this figure legend, the reader is referred to the web version of this article.)

The conservation scores for the hSARSr-CoVs and the SARSr- and MERSr-CoVs were mapped onto the S monomeric structure (SARS-CoV-2 structure, PDB entry 6VYB) as illustrated in Fig. 1.

Additionally, we have identified conserved clusters (CC) (≥10 continuous residues) on the S S1 and S2 subunits of hSARSr-CoVs and between SARSr- and MERSr-CoVs (Table 2; Figure S-1). High level of conservation was also found between SARS-CoVs, SARS-CoV-2 and bat-SL-CoVs, with CC overlapping the ones described for hSARSr-CoVs (Table 2).

**Table 2:** **Summary of the conserved regions identified in the S1 and S2 subunits of the Spike protein.** Only conserved clusters comprised by a minimum of 10 aa and with a conserved score ≥ 7 were considered for either the hSARSr-CoVs, the SARSr-CoVs (including the bat-SL-CoVs) and the SARSr- and MERSr-CoVs. Regions common to all three groups are highlighted in bold and blue.

**human SARSr-CoV**						**SARSr-CoV**						**SARSRr- and MERSr-CoV**					
**S1 subunit**			**S2 subunit**			**S1 subunit**			**S2 subunit**			**S1 subunit**			**S2 subunit**		
**Region**	**Position**	**Lenght (aa)**	**Region**	**Position**	**Lenght (aa)**	**Region (analogue to the hSARSr-CoVs)**	**Position**	**Lenght (aa)**	**Region**	**Position**	**Lenght (aa)**	**Region (analogue to the hSARSr-CoVs)**	**Position**	**Lenght (aa)**	**Region**	**Position**	**Lenght (aa)**
**NTD.1**	T51-T63	13	**NTD.1**	S691-E702	12	**NTD.1**	T51-T63	13	**S2-NTD.1**	S691-E702	12	**NTD.4**	**V308-V320**	**13**	**sv-CR.1**	**I818-K835**	**18**
**NTD.2**	T95-G107	13	**NTD.2**	M740-C749	10	**NTD.2**	T95-G107	13	**S2-NTD.2**	M740-C749	10	**RBD.3**	**S373-L390**	**17**	**sv-CR-HR1.1**	**Y904-Q913**	**10**
**NTD.3**	T274-C291	18	**NTD.3**	N751-L767	17		A262-P272	11	**S2-NTD.3**	N751-L767	17	**SD1.1**	**C538-T553**	**16**	**sv1-HR1-CH.1**	**L966-S975**	**11**
**NTD.4**	**V308-V320**	**13**	**FP.1**	F797-P809	13	**NTD.3**	T274-C291	18	**FP.1**	F797-P809	13	**SD2.4**	**S659-A672**	**14**	**sv2-HR1-CH.1**	**E990-L1001**	**12**
**RBD.1**	I326-F347	22	**CR.1**	**K814-F833**	**20**	**NTD.4**	**V308-V320**	**13**	**CR.1**	**K814-F833**	**20**		–	–	**sv3-HR1-CH.1**	**T1006-A1015**	**10**
**RBD.2**	R355-A372	18	**CR.2**	K835-I844	10	**RBD.1**	I326-F347	22	**CR.2**	K835-I844	10		–	–	**sv1-CD-HR2.1**	**S1147-N1158**	**12**
**RBD.3**	**F374-S383**	**10**	**CR.3**	A846-A871	26	**RBD.2**	R355-A372	18	**CR.3**	A846-A871	26		–	–	**sv2-CD-HR2.1**	**K1211-M1229**	**19**
**RBD.4**	N394-G416	23	**CR-HR1.1**	**G885-K921**	**37**	**RBD.3 | RBD.4**	**F374-G416**	**43**	**CR-HR1.1**	**G885-N928**	**43**						
**RBD.5**	I418-F429	12	**HR1-CH.1**	**A944-Q1054**	**111**	**RBD.5**	I418-F429	12	**HR1-CH.1**	**A944-Q1054**	**111**						
**RBD.6**	G502-L518	17	**CH.1**	A1056-P1069	14	**RBD.6**	G502-L518	17	**CH.1**	A1056-P1069	14						
**SD1.1**	**S530-T553**	**24**	**CH-CD.1**	Q1071-V1096	26	**SD1.1**	**L533-T553**	**21**	**CH-CD.1**	Q1071-V1096	26						
**SD2.1**	P589-S605	17	**CD-HR2.1**	**P1112-I1232**	**121**	**SD2.1**	P589-S605	17	**CD-HR2.1**	**P1112-I1232**	**121**						
**SD2.2**	A623-G639	17	**HR2-CP.1**	L1234-G1246	13	**SD2.2**	A623-G639	17	**HR2-CP.1**	L1234-G1246	13						
**SD2.3**	N641-N657	17	**CP.1**	C1248-T1273	26	**SD2.3**	N641-N657	17	**CP.1**	C1248-T1273	26						
**SD2.4**	**S659-T676**	**18**	–	–	–	**SD2.4**	**S659-T676**	**18**	–	–	–						

The hSARSr-CoV S1 subunit contains 15 CC, named according to the dominant domain of the clustered residues (listed in Table 2). The longest S1 CCs consist of RBD.1, RBD.4 and SD1.1. The CCs RBD.3 and RBD.5 exhibit a conservation score of > 10.5 for the hSARSr-CoVs and the NTD.4, RBD.3, SD1.1 and SD2.2 are also conserved among all beta-CoVs included in the study.

The hSARSr-CoVs S2 subunit comprises 14 CC (Table 2). The CC S2-NTD.2, FP.1, CR.1, CR-HR1.1, HR1-CH.1, CH.1, heptad repeat 2-cytoplasmic domain.1 (HR2-CP.1) and CP.1 exhibit a conservation score of >10.5 for the hSARSr-CoVs. The longest S2 CCs consist of HR1-CH.1 and connector domain (CD)-HR2.1. Some of these CC are concurrently identified as shorter variant (sv) forms, when comparing hSARSr-CoVs and between SARSr- and MERSr-CoVs. Specifically, a shorter variant of CR.1 (sv-CR.1) and CR-HR1.1 (sv-CR-HR1.1), three shorter variants of HR1-CH.1 (sv1-HR1-CH.1, sv2-HR1-CH.1 and sv3-HR1-CH.1), and two shorter variants of CD-HR2.1 (sv1-CD-HR2.1 and sv2-CD-HR2.1) are found among all beta-CoVs included in the study.

Taken all together, high level of conservation was found in several domains of S1 and S2 subunits of hSARSr-CoV S, with a similar number of CC found in each subunit. The majority of the S1 CC are located in the RBD, while the S2 subunit comprises longest CC and mostly located in the protein regions that encompasses the CR, HR1, CH and HR2 domains. The average degree of protein sequence identity for some of these S2 CC is approximately 100%, which corresponds to almost absolute conserved sites (cs > 10.5).

In regard to SARSr- and MERSr-CoVs, 4 CC were found in the S1 subunit and 7 CC were found in the S2 subunit. The largest pocket within S1 lies in the RBD and corresponds to the RBD.3 described for hSARSr-CoVs. The majority of the S2 CC are located in the protein regions that comprises the domains CR, HR1 and CH.

Overall, this suggests that the S1 subunit exhibits a slightly highest conservation (99.8%) when compared to S2 (95.6%) in SARS-CoV-2. However, for hSARSr-CoVs and when comparing SARSr- and MERSr-CoVs, S2 subunit presents a higher level of conservation.

A good antiviral target will have a structure predominantly conserved, with advantageous structural/druggable features, and concomitantly displays an important function regarding the virus replication cycle [31], [32]. We identified the S1-RBD and the regions that include the CR, HR1 and CH in the S2 subunit, as the most promising domains and further explored them for druggability.

### 2.3 Druggability studies

Only pockets comprised of a minimum of 10 aa and defined with a druggability score ≥ 0.4 were considered (as described in the Materials and Methods section), based on the premise that a minimal pocket size is required in order to indulge a proper interaction between the target and potential ligands. Pockets with an impaired ability to bind a ligand are named decoy pockets and frequently include small cavities comprised by less than 10–14 residues [33], [34]. Druggable pockets are required to bear other advantageous structural features which can be estimated/characterized using pocket descriptors that include the volume, depth, enclosure, solvent-accessible surface area, buriedness of residues in the interface, etc [35].

In the S pre-fusion state, three monomers intertwine to form a trimer complex which represents the predominant conformation of the S protein in vivo [6]. The binding of the S protein to the receptor promotes a conformational switch of the S1-RBD from down to up conformation (determining S closed and open states). This results in a dissociation of the S1-ACE2 complex and S1 monomers from the pre-fusion protein and prompts the conformational transition to the post-fusion state. The specific mechanism, and the range of S conformational changes and states, remain uncharacterized. [36], [37], [38]. Several studies have shown that the ectodomain and the RBD expression in eukaryotic systems are stable and appear in the monomer conformation in high yield; and that soluble ACE2 binds to both monomer and trimer conformations [37], [39], [40], [41], [42]. In this context, to fully characterize the S protein, it is important to explore both the monomer and trimer in structural studies. A comprehensive analysis of the full-length S monomer (in both open and closed conformations) is discussed in a section of the Supplemental Data.

#### 2.3.1 Spike-RBD

The S-RBD has been repeatedly emphasized by several authors as bearing a higher antiviral target potential [28], [29], [30], and, for that reason, we describe it in more detail. Sixteen druggable pockets were identified in the three S-RBD structures (from a total of 29) for the DoGSiteScorer (DGSS) server. Most pockets are shared by all the RBD structures, since no major conformational differences exist among them. The supplement Table S-4 displays the 16 pockets identified for each structure, along with the pocket descriptors: size, volume and and druggability score.

**Fig. 2:**
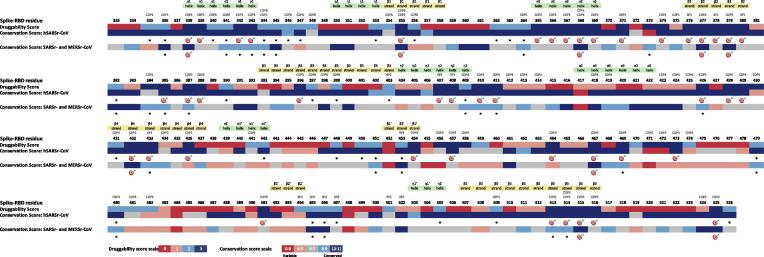
**Overall alignment of Spike-RBD druggability along with the conservation scores for each residue position.** The druggability prediction was based on the descriptors algorithm of each pocket bioinformatics tool: SF, DGSS and PDS. The potential conserved druggable sites/residues are marked with an asterisk and the T-RHS are marked with a target. The conserved druggable pockets allocated to each site/residue along with the secondary structure elements are indicated at the top of the picture.

A linear schematic representation of the S-RBD containing the druggability information for each aa position is shown in Fig. 2. Differences in the distribution of druggable aa regions/residues within the RBD, may help to distinguish which ones have a potential role in RBD function or structure. The conserved druggable regions and the top-ranked hot spots (T-RHS) identified through comparative analysis of the S-RBD druggability and conservation scores in both hSARSr-CoVs and SARSr- and MERSr-CoVs are depicted in Fig. 2.

Some of these conserved druggable regions are located close together and can form larger pockets, named as consensus druggable pockets (CDP). Six CDP (common to all RBD structures) were characterized based on the comparative druggability study. The secondary structure elements were predicted based on the ESPript, as previously performed by Wang *et al*. [43], [44]. Three out of these 6 CDP have more than 14 aa and, interestingly, one of the CDPs is not found in the most complex structures (monomer or trimer). The supplemental Table S-2 displays the CDPs identified for the S-RBD (along with the aa number and composition).

**Fig. 3:**
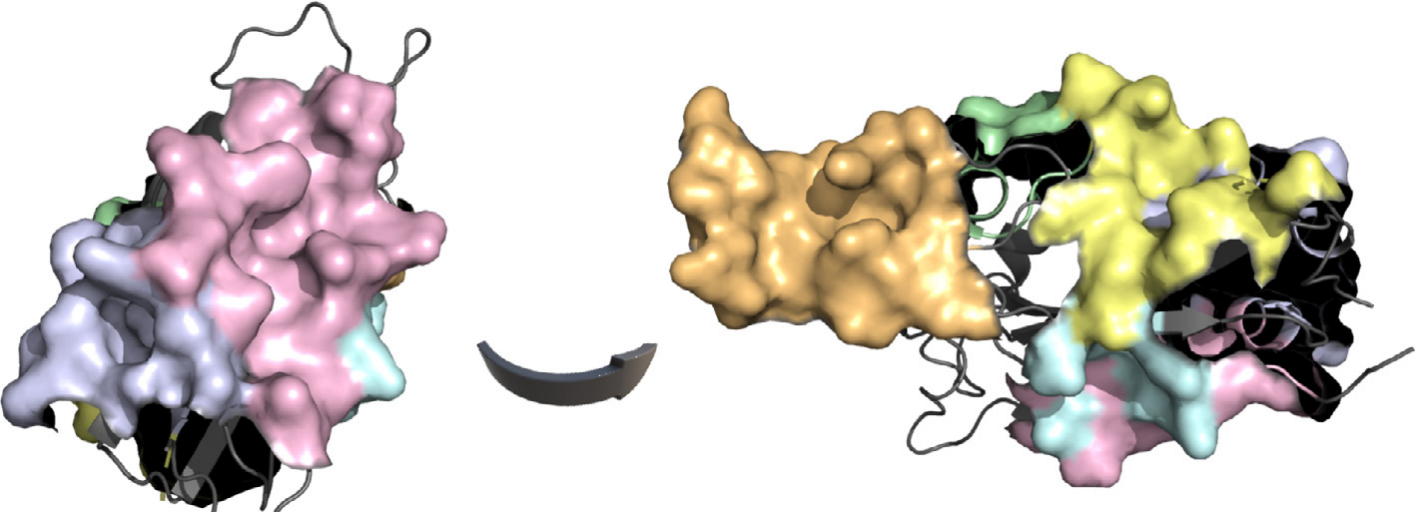
**Mapping results of the highest-ranked consensus druggable pockets onto the Spike-RBD crystallographic structure of SARS-CoV-2 (PDB: 6VW1)**. The top-ranked pockets: CDP1 to CDP5 are highlighted in shades of pink, pale blue, orange, green, yellow and cyan, respectively. The number and aa composition of each CDP are shown in Supplemental Table S-2. The figures were produced with PyMOL Molecular Graphics System [101]. (For interpretation of the references to colour in this figure legend, the reader is referred to the web version of this article.)

The CDP1 (helices α1-α2, α1′ and strands β4-β5) is comprised of 16 residues. The CDP2 (helix α2 and strands β2, β4 and β5) comprises 15 residues. The CDP3 (strands β1′ and β2′), only found in S-RBD structures, contains 14 residues. The CDP4 (helix η2 and α3) is comprised by 11 residues; the same number for the CDP15 (strands β1, β3-β5). The CDP6 (helix α1; strands β1 and β3) contains 12 residues Two small pockets (SP1 and SP2) comprised by 8 residues each were found in all RBD structures. The spatial arrangement of the highest-ranked CDPs for the S-RBD based on the DGSS algorithm is shown in Fig. 3.

The 43T-RHS for hSARSr-CoVs and 9 for SARSr- and MERSr-CoVs (out of 75 and 35 conserved druggable residues - CDR, respectively) are listed in Table S-3 and depicted in Fig. 2; and demonstrate that the S-RBD bears a high potential of druggability. CDR and T-RHS have been identified for hSARSr-CoVs and for SARSr- and MERSr-CoVs, establishing S-RBD as a promising anti-CoV target for the design and development of antiviral and vaccine strategies.

#### 2.3.2 Spike trimer conformation

The homotrimeric S protein is suggested to adopt two dynamic distinct states (referred to as closed and open conformation). In the S trimer analysis, both closed and open state conformations along with an asymmetrical homotrimer, in which a single RBD domain is featured in the up conformation (semi-open state), were included.

A total of 170 potential druggable pockets (out of 304) were identified in both open (61 pockets), semi-open (67 pockets) and closed (42 pockets) conformations, for the DGSS server. Several pockets are shared between the three structures, independently of the RBD conformation. Moreover, it is possible to identify pockets common to the CDPs previously predicted for the S monomer structures, however some are exclusively found when the S protein assembles into a homotrimeric complex. The supplemental Table S-5 displays the pockets identified for each S trimer conformation, along with the pocket descriptors: size, volume and druggability score.

Similarly, several pockets identified in the trimer are also shared by all structures, independently of the conformational RBD switch within the structure. The CDPs previously identified for the S monomer (CDP.M) maintain advantageous structural features regarding the druggability prediction in the trimeric structures (CDP.T). None was found to be surface-inaccessible and the residues were not found to be buried in the interface when the monomer subunits combine to form a trimeric complex, thus, all CDPs identified for the S monomer group are represented in the trimer.

28 CDPs (22 CDP with ≥ 14 aa) were found in S trimer structures, 12 in S1 (CDP1T-S1 to CDP12T-S1), 11 in S2 (CDP1T-S2 to CDP11T-S2) and 5 incorporate both S1 and S2 (CDP1T-S1S2 to CDP5T-S1S2). The CDPs identified for the S trimer can be composed of one, two or the three monomer subunits of the trimeric complex. Some of these CDPs have not been identified in the monomer analysis, suggesting that the trimeric structure contributes to expand and improve the druggability potential of the protein. Thus, a higher number of CDPs (and also T-RHS and CDR, as described below) can be found in the trimer conformation in comparison to S monomer.

Four S1 CDPs (CDP3T-S1, CDP5T-S1, CDP9T-S1 and CDP11T-S1), located mainly in the RBD, can only be found in the trimeric structure. In the same vein, 7 pockets identified within the S2 subunit (CDP2T-S2, CDP4T-S2, CDP5T-S2, CDP6T-S2, CDP8T-S2, CDP9T-S2 and CDP11T-S2) and all the 5 CDPs which incorporate both S1 and S2 subunits (CDP1T-S1S2 to CDP5T-S1S2) are only represented in the trimer. The supplemental Table S-2 displays the CDPs identified for the S trimer conformation (along with the aa number and composition; and the corresponding location/domain).

The 22 CDPs shared by all trimeric structures and exhibiting ≥ 14 aa have been analyzed according to the sequence location within the protein (from the N- to the C-terminal). In regard to the S1 subunit, the first two CDPs (CDP1T-S1 and CDP2T-S1) are located in the S1-NTD and are very similar in structure and aa composition to the CDP1M-S1 and CDP2M-S1, respectively, described for the monomer conformation. The CDP3T-S1 (in the NTD and RBD) presents as a larger CDP that resembles a combination of both CDP3M-S1 and CDP11M-S1. The CDP4T-S1 (in the NTD) is analogous to the CDP5M-S1. The CDP5T-S1 (in the NTD and RBD) is only found when two monomer subunits bind together. The CDP6T-S1 (in the NTD) and the CDP7T-S1 (in the RBD) are analogous to the CDP6M-S1 and CDP8M-S1, respectively. The CDP9T-S1 and CDP11T-S1 (in the RBD) are only described for the trimeric structure. The CDP12T-S1 (in the NTD) is analogous to the CDP14M-S1.

In regard to the S2 subunit, the CDP1T-S2 is analogous to the CDP1M-S2 described for the monomer structure. A larger pocket composed of 163 residues, CDP2T-S2, is only found in the trimeric structure when three monomers bind together; and is similar in composition to the CDP1T-S2 and the analogous pockets CDP3M-S2 and CDP9M-S2. The CDP3T-S2 is analogous to the CDP4M-S2. The CDP8M-S2 described in the monomer analysis can be found within the trimeric structure in a more complex (recruiting ≥ two monomer subunits) larger pocket: (1) either together with the CDP6M-S2 to form the CDP4T-S2; or (2) together with both CDP6M-S2 and CDP7M-S2 to form the CDP5T-S2. The CDP6T-S2 (in the S2-NTD and CH) is only found in the trimeric structure when two protomers bind together. The CDP7T-S2 is analogous to the CDP2M-S2. The CDP8T-S2 (in the S2-NTD, FP, HR1, CH and CD) is only identified in the trimeric structure. The CDP9T-S2 (mainly located in the HR1, CH and CD) is described only in the trimeric structure. The CDP10T-S2 is analogous to the CDP7M-S2.

In addition to the CDPs described for S1 and S2, the trimeric structure bears CDPs that include both subunits. The CDP1T-S1S2 (of 40 residues) is in part similar to the CDP3M-S2 described for the monomer structure (located in the S2-NTD, HR1 and CH) but is also composed of additional residues pertaining to the S1-NTD. The CDP2T-S1S2 (of 71 residues) is formed by a pocket pertaining to two protomers binding together, one analogous to the previously described CDP1T-S1S2 and the other composed of residues that form both the CDP12T-S1 and the CDP13M-S1. The CDP3T-S1S2 (of 63 residues) is in part analogous to the CDP7M-S1 (in the RBD); while it also comprises additional residues belonging to the SD1 and S2-HR1. The CDP4T-S1S2 is located in the S1-RBD and S2-CH. The CDP5T-S1S2 (of 42 residues) is also formed by a pocket pertaining to two protomers binding together, one (19 aa) located in the S1-NTD and SD2 and the other (23 aa) located in the S2-NTD.

**Fig. 4:**
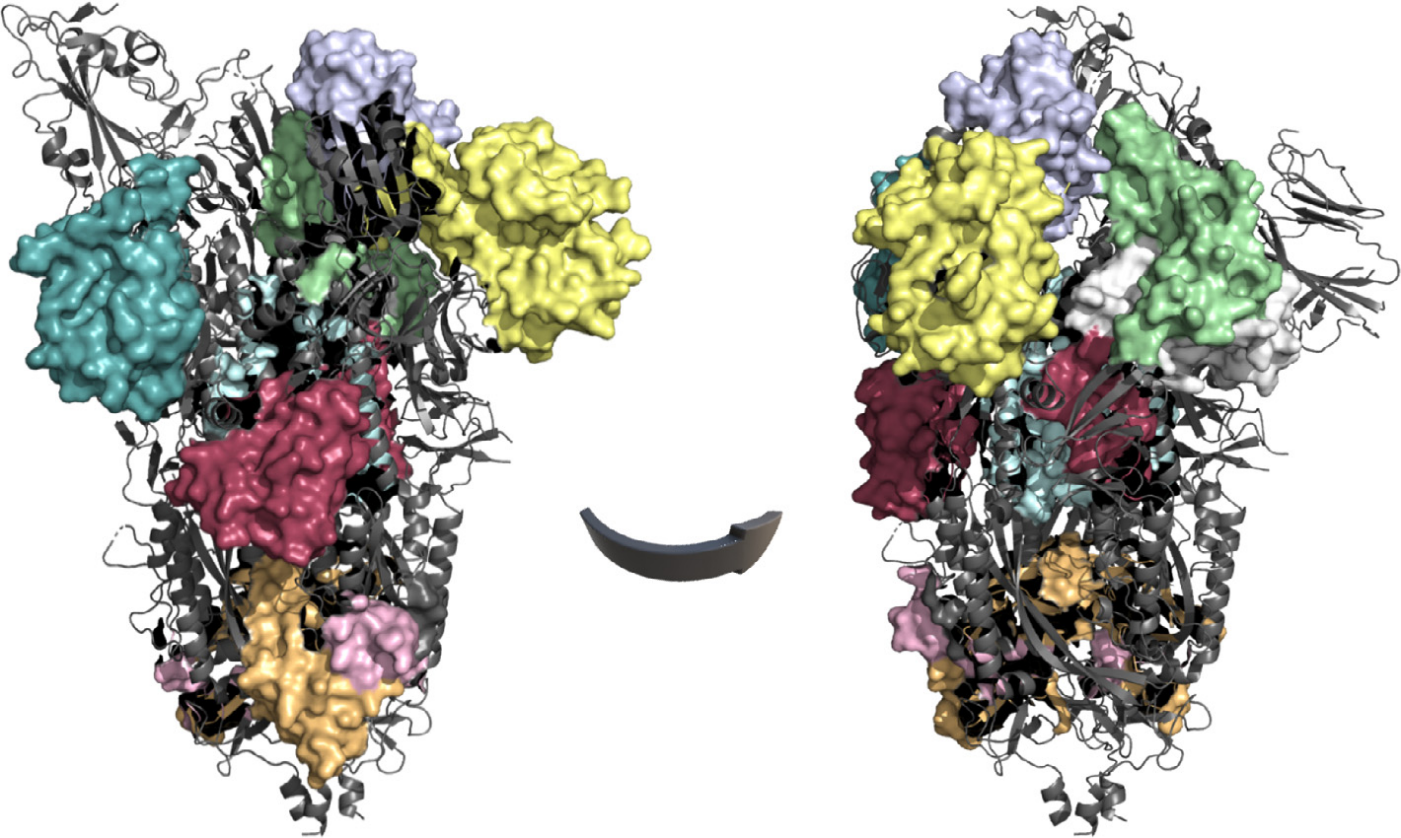
**Mapping results of the highest-ranked consensus druggable pockets onto the Spike trimer crystallographic structure of SARS-CoV-2 (PDB: 6VYB)**. The top-ranked pockets: CDP5T-S1, CDP6T-S1, CDP9T-S1, CDP2T-S2, CDP4T-S2, CDP8T-S2, CDP2T-S1S2, CDP3T-S1S2, CDP5T-S1S2 are highlighted in shades of yellow, turquoise, pale blue, cyan, orange, pink, grey, green and raspberry, respectively. The number and aa composition of each CDP are shown in Supplemental Table S-2. The figures were produced with PyMOL Molecular Graphics System [101]. (For interpretation of the references to colour in this figure legend, the reader is referred to the web version of this article.)

Although the analysis of the differences between S state conformations is out of the scope of this article, two main differences are worth to mention. First, a pocket of 20 residues located in the S1-NTD and RBD (aa 117, 128–130, 167–170, 229–231, 357, 359–360, 393–394, 520–521, 523), with no expression in the closed S conformation, is found in the open S conformation structures. Second, a large pocket (in the RBD) comprised of the combination of CDP3T-S1, CDP8T-S1, CDP9T-S1, CDP10T-S1, CDP11T-S1, CDP3T-S1S2, CDP4T-S1S2 and SP2T-S1 is only found in the open conformation structure. Taken together, we suggest that, for the trimeric complex, the open conformation bears additional potential binding regions within the RBD for distinct modes or types of S-RBD interactions. The spatial arrangement of the highest-ranked CDPs for the trimer conformation is shown in Fig. 4.

**Fig. 5:**
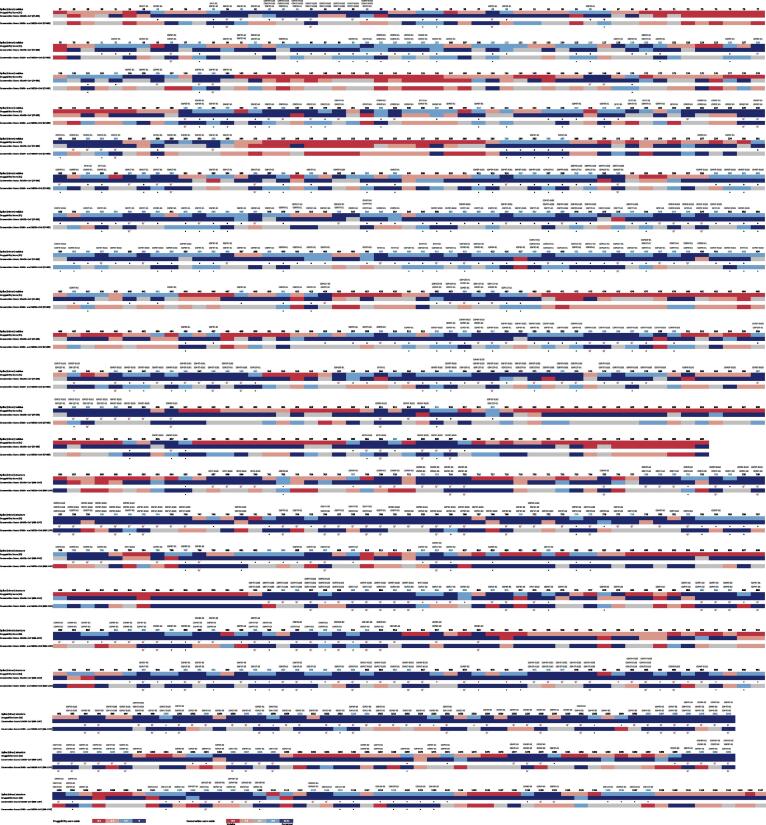
**Overall alignment of Spike S1 and S2 druggability along with the conservation scores for each residue position.** The druggability prediction was based on the descriptors algorithm of each pocket bioinformatics tool: SF and DGSS; and for all the predicted S state conformation (open, semi-open and open). The potential conserved druggable sites/residues are marked with an asterisk and the T-RHS are marked with a target. The conserved druggable residues shared by the S monomer and trimer conformations for the hSARSr-CoVs are coloured in blue; and the conserved druggable pockets allocated to each site/residue are indicated at the top of the picture. (For interpretation of the references to colour in this figure legend, the reader is referred to the web version of this article.)

The last stage of the study consisted of a global comparative analysis of the S trimer druggability together with the conservation scores for the hSARSr-CoVs and the SARSr- and MERSr-CoVs (Fig. 5).

The 274T-RHS for drug targeting (out of 553 CDR) identified in the hSARSr-CoV S trimer comprise 99 hot spots in S1 (out of 288 CDR); and 175 hot spots (out of 265 CDR) in S2. The supplemental Table S-3 displays the CDR and T-RHS identified for the S trimer conformation*.* Comparing the two subunits, no major differences were found in the absolute number of CDR. S1 displays a slightly higher number of CDR compared to S2 (S1: 288 vs S2: 265). However, most of the T-RHS are located within S2, with a prevalence of 29.8% (175/588), whereas S1 shows a prevalence of 14.4% (99/685). It has been found that about 42% of the CDR in S1 are located in the RBD. The RBD is the most druggable domain within the S1 subunit (120 CDR and 33T-RHS out of 195 overall residues), followed by the SD1 (with 38 CDR and 19T-RHS of a total of 94 residues). The SD2 is the least relevant domain within the S1 subunit in regard to the druggability potential, as previously predicted in the monomer analysis. For the S2 subunit, it is worth to mention that 12 CDR were found within the FP (total length of 19 aa); and CH was identified as the most conserved druggable region (65 CDR and 50T-RHS in a 99-aa domain length) although the FP, S2-NTD, HR1 and CR are also promising druggable regions (in decreasing ranking order).

The 110T-RHS for drug targeting (out of 164 CDR) found in the SARSr- and MERSr-CoVs comprise 28 hot spots (out of 160 CDR) in the S1 subunit and 82 hot spots (out of 132 CDR) in the S2 subunit (Table S-3). In the same vein to that observed for the hSARSr-CoVs, S1 displays a higher number of CDR compared to S2 (S1: 160 CDR vs S2: 132 CDR), while most of the T-RHS are located within S2 (S1: 28 vs S2: 82). The majority of the S1 CDR are located in the NTD, RBD and SD1. The SD1 displays 20 CDR and 5T-RHS out of 64 residues that comprise the domain. Although the RBD was found to be the most druggable domain in hSARSr-CoVs, it ranks second in the SARSr- and MERSr CoV group, with some regions within the RBD showing lower conservation among all four beta-CoVs. The S1-SD1 is the most conserved druggable domain for SARSr- and MERSr-CoVs, followed by the RBD. For the S2 subunit, a different pattern is evident when compared to hSARSr-CoVs. Most of the CDR or T-RHS are found in the CH, followed by the HR1 and CR. The FP displays a lower conservation score among all CoV species and does not represent a conserved druggable domain in the SARSr- and MERSr-CoV group. The CH is the most conserved druggable domain in the S2 subunit, for this group.

The comparative analysis of the S monomer and trimer conformations highlighted that 171 S1 CDR and 119 S2 CDR are shared by these conformations, in regard to the hSARSr-CoVs. Moreover, 95 S1 CDR and 59 S2 CDR are present in both monomer and trimer conformations in SARSr- and MERSr-CoVs (Table S-6). The CDR shared by the monomer and trimer conformations are highlighted in blue in Fig. 5.

### 2.4 Contribution of the findings to previous research

To the best of our knowledge, there are no published studies to date addressing and comparing the conservation and druggability of the CoV S protein, for such a wide range of sequences (n = 1086 S1; n = 1096 S2) from four Beta-CoVs (SARS-CoV-2, SARS-CoVs, MERS-CoVs and Bat-SL-CoVs) and the crystallographic structures of all available SARS-CoV-2 S proteins (S-RBD, monomer and trimer structures in either closed, semi-open and open state conformations, when applied). In the majority of recent studies, a comparative analysis of the S protein has been performed with one reference strain for each CoV type and thereby taking into account only the most prevalent residue harbored at a given position; It does not represent diversity and it overestimates the protein conservation score. To overcome this, we performed the conservation analysis using the total number of protein sequences treated for each CoV type, so that the conservation estimation takes into account the variations in the aa composition within each CoV.

The present study has revealed the most propitious S domains to target in regard to the conservation and druggability analysis of both S monomer and trimer conformations. The S1-RBD represents a promising anti-COV target and is the most conserved druggable domain in the monomer analysis for hSARSr-CoVs and for SARSr- and MERSr-CoVs; and in the trimer analysis for hSARSr-CoVs. For the SARSr- and MERSr-CoV trimer, the RBD ranks second after the SD1 domain; which is concordant with different CoVs using distinct host receptors for entry [45]. The SD1 stands as the most conserved druggable domain among all four Beta-CoVs analyzed. In this context, both RBD and SD1 domains should be further addressed in future studies that target the S1 subunit.

In regard to the S2 subunit, the FP was found to bear a high potential of druggability exclusively in hSARSr-CoVs (its conservation degree decreases when considering other beta-CoV species). On the other hand, the CH demonstrates a higher conservation-druggability potential among hSARSr-CoVs and in SARSr- and MERSr-CoVs and is the most conserved druggable domain within the S2 subunit. Other S2 domains, such as the CR and HR1, are alternative potential antiviral targets and can also be considered in anti-CoV strategies.

We have demonstrated that regardless of the S protein conformation states, highly conservation regions among either the hSARSr-CoVs and the SARSr- and MERSr-CoVs can overlap with potential binding sites/residues, rendering the Spike protein a suitable antiviral target.

Our computational analysis has revealed specific T-RHS and CDR and the corresponding conserved druggable pockets within each domain of the S protein, in hSARSr-CoVs and SARSr- and MERSr-CoVs. The majority of the T-RHS identified in our study, in regard to the S-RBD analysis, lie at the core structure of this domain rather than at the receptor-binding motif (35/43 in hSARSr-CoVs; 8/9 in SARSr- and MERSr-CoVs). Recently, Wang et al. have shown that SARS-CoV-2 and/or SARS-CoV neutralizing human monoclonal antibodies 47D11 and CR3022 preferentially target the RBD core domain [46], which supports our findings. Additionally, Pinto et al. identified a human monoclonal antibody S309 with neutralizing activity against the SARS-CoV-2, which mainly targets the RBD epitope N343 [47], which is also a potential hot spot for drug targeting identified in our study. Of the S protein targeted B- and T-cell epitopes that are promising candidates for vaccine design against SARS-CoV-2 [48], [49], 40B-cell and 44T-cell were identified as T-RHS or CDR residues in our analysis.

The CDR shared by the S monomer and trimer structures: C336, C361, C379, C391, C432, C525 (RBD), Q920 N955 (HR1) have been previously described in the literature for their role in protein structure [12], [50]. The cysteine residues found in the RBD can form pairs of disulfide bonds (C336–C361, C379-C432 and C391–C525) that help to stabilize the β sheet structure of this domain [12]. Additionally, Lan et al. revealed a total of 17 residues from the SARS-CoV-2 RBD that are in close contact to the ACE2 receptor, but, of these, only Y453 and Y505 are highlighted by our study as trimer CDR for drug targeting [12].

Two S protein motifs within the S2 subunit have been studied by other authors. The motif KRSFIEDLLFNKV (aa 814–826), located within and flanking the S2′ cleavage site (underlined R), is suggested to be required for virus activation and cell entry [51]. Four T-RHS or CDR identified here are among the 13 residues that comprise this motif, namely: Q814, R815 (S2′ cleavage site), S816 and F823. Also, the 3C-like proteinase cleavage site/region TGRLQ^SLQTY (aa 998–1007), located at the S2-CH domain, is currently being studied. The majority of the residues that comprise this motif (9 out of 10) constitute T-RHS or CDR identified in our study, in particular T998, G999, R1000, L1001, Q1002, S1003, Q1005, T1006 and Y1007 [52].

The trimer-binding interface between individual S protomers and the interaction sites of trimerization are not fully characterized for the SARS-CoV-2. Recent studies have suggested several residues that contribute to the formation or to stabilize the S trimeric structure; and most of them are located at the S2 subunit (mainly in the S2-NTD, FP, CR, HR1, CH and CD) [53], [54], [55], [56], [57], [58]. Thirteen of these residues (E702, Y707, N709, N710, Y789, K790, K795, F797, G798, T859, G891, Q895, F898) are described by multiple authors and represent CDR or T-RHS for drug targeting highlighted in our study [53], [54], [55], [56], [57], [58]. The main CDPs that incorporate the residues in the trimer-binding interface consist of: CDP3T-S2, CDP4T-S2, CDP5T-S2, CDP8T-S2, CDP9T-S2. Additionally, Peters et al. identified 3 residues (A520, P521 and A522 – highlighted as CDR in our study) that play a role in stabilizing the RBD through interactions with the NTD of the adjacent protomers [57]. The key residues that contribute to S trimerization can be potentially modulated by therapeutic agents in order to disrupt the quaternary structure assembly of the protein.

*In silico* studies suggest that the SARS-CoV-2 S S1 might potentially bind to the human MERS-CoV receptor dipeptidyl peptidase-4 (DPP4, also known as CD26) [52]. Vankadari et al. predicted 14 residues within the RBD that may lie in the S1:CD26 interaction interface, but only 5 of these residues (R408, Q409, D467, S469, P491) are considered CDR based on the criteria defined in our study [52]. Furthermore, the toll-like receptor 4 (TLR4), which is involved in the recognition of molecular patterns and mediate inflammatory responses, may also interact with the S protein via 10 residues located in the S1 subunit. Three of these residues were identified in this study as T-RHS residues, namely Y204, V289 (S1-NTD) and F562 (SD1). Both the S:CD26 and S:TLR4 interactions may constitute potential alternative broad antiviral targets [59].

It is established that SARS-CoV-2 S protein enters into the host cell through the ACE2 receptor; but it also uses sialic acids linked to gangliosides at the host plasma membrane, which may improve the virus attachment to lipid rafts and facilitate the contact with the ACE2 receptor [60]. Fantini, et al. have identified a ganglioside-binding domain at the S1-NTD of S protein (aa 111–162) [60]. The residues S116, I119, V120, N121, V126, I128, F133, C136, P139, F140 comprise the ganglioside-binding domain and were identified in our study as potential T-RHS or CDR for drug targeting. Moreover, chloroquine, and its close structural analogues, bind sialic acids and gangliosides with high affinity and have shown to block the S:ganglioside interaction [60]. The authors also suggested that the azithromycin might interact with this ganglioside-binding domain within the S protein [61].

Drug repurposing or the chemical optimization of existing drugs represent an effective drug discovery approach which has the potential to reduce the time and costs associated to the *de novo* drug discovery and development and the subsequent clinical trials process [62]. *In silico* and *in vitro* studies have recently demonstrated that the arbidol (anti-influenza inhibitor) and the nelfinavir mesylate (anti-HIV inhibitor) can potentially inhibit the SARS-CoV-2 replication [63], [64]. The majority of the key residues involved in the arbidol:S interaction (7 out of 9 residues) have been identified here as potential hot spots, in particular: E780, K947, E1017, R1019, S1021, L1024, T1027 (S2-NTD, HR1, and CH domains). In regard to nelfinavir, Musarrrat et al., have shown that the following CDR may lie in the interface of S:nelfinavir interaction: Q954, Q957, A956, L1012, I1013 (FP and HR1 domains) [64]. Other docking assays have suggested that the CDR: R319 (S1-NTD), C391, L517 (RBD), C538, F543, N544, Q564, P589 and S591 identified in this study represent active site residues that potentially interact with several medicinal compounds such as arzanol, genistein, resveratrol, rosmanol or thymohydroquinone [65].

The HR1 has also been identified in our study as a promising conserved druggable region. Based on the finding that HR1 and HR2 regions are able to interact with each other to form a 6-helical bundle – essential for viral and cell membrane fusion – several authors have reported HR1- and HR2-derived peptides that can inhibit this fusion [66], [67], [68], [69], [70], [71]. Specifically, the fusion inhibitors HR2P, EK1, and EK1C4, exhibited a broad inhibitory activity against the SARS-CoV-2 and other SARSr- and MERSr-CoVs [66], [67], [68], [69], [70], [71].

Additionally to the T-RHS and CDR described in the literature, we identified new potential hot spot residues which, to the best of our knowledge, have not been described before, regarding its druggability, structural importance and/or individual role in SARS-CoV-2. These include 181 (66%, 181/273) and 72 (65%, 72/110) residues identified in the S trimer structure of hSARSr-CoVs and SARSr- and MERS-CoVs, respectively. In both groups, these new potential hot spots lie essentially at the S2 subunit of the protein, particularly at the S2-NTD and CH domains (residues listed in Table S-7).

The potential hot spots residues are mostly surface exposed, within pockets of large volume and size, high enclosure and depth. They may represent advantageous targets for molecular and pharmacological modulation, since they potentially establish key interactions with host receptors or other molecules, or might play other roles in receptor recognition, S trimerization, S processing or in the mechanism of RBD conformational change. Moreover, the S protein represents a target for antibody-mediated neutralization by the host immune response and, consequently, constitutes a major antigenic component for structure-based vaccine design.

In this context, it is important to consider the effect and implications of the glycan shielding on immune evasion. Viral glycosylation not only mediates molecular recognition events, protein stability and folding, but it also plays a role in masking immunogenic protein epitopes from the host humoral immune system [72], [73]. This could, in turn, hamper the neutralizing antibodies development. Several authors have recently predicted the SARS-CoV-2 S glycan shield pattern [52], [72], [73], [74]. Casalino et al. characterized the glycosylation profile of the S protein and found that the HR2, TM and CT domains may be inaccessible to large molecules such as antibodies [72]. In this context, only small molecules should be explored when considering future antiviral strategies targeting these regions. On the contrary, the S head portion (including the S1-RBD, -SD1; SD2-FP, -CR, -HR1 and -CH domains, which are highlighted in our study) represents a more promising target, either for small molecules or for larger peptide and antibody-size molecules, since it is less shielded by the glycans [72]. Additionally, other authors suggest that the SARS-CoV-2 S protein is less densely glycosylated and, consequently, with a more vulnerable glycan shield when compared to other viral glycoproteins (HIV-1 envelope protein, Lassa virus glycoprotein complex and the influenza hemagglutinin) [73], [74]. This may represent advantageous features in order to elicit SARS-CoV-2-neutralizing antibodies against the hot spots identified in our study. These authors have also identified glycosylation residues (N165 and N234) that may play a structural role in stabilizing the S1-RBD in the up conformation (determining the S open state) [72], [73]; and the residue N234 represents a potential hot spot for drug targeting identified in our study.

The comprehensively structural characterization performed in our study should prompt the application of rational structure-based virtual screening, molecular docking and other in silico-chemico-biological approaches for the identification of potential novel CoV chemical inhibitors or monoclonal antibodies/vaccines. Although the computational strategies have the potential to accelerate the drug discovery process and guide the posterior experimental approaches, they have limitations considering the experimental knowledge gaps [75], [76]. Therefore, these potential hot spots should be experimentally studied *in vitro* and in vivo regarding its individual role in protein function or structure among beta-CoVs circulating in the human population. These data may also endorse the evaluation of SARS-CoV-2 S mutations resulting from the evolutionary adaptation of the virus to the human host.

The most relevant hot spot residues can be explored in discovery, design or development of chemical compounds or pan-Beta-CoV monoclonal antibodies that have the potential to inhibit the predicted pockets. Priority should be given to hot spots residues allocated to one or multiple consensus druggable pockets within the most promising S domains (and common to all S states). In this vein, a molecule designed to target one of these CDP could potentially interact with multiple sites/residues within the same CDP, or inhibit sites/residues that integrate multiple CDP within a major pocket. Consequently, other residues belonging to the considered CDP could be further studied in order to optimize or potentiate additional inhibitor-pocket binding interactions. A multi-target inhibitor can be, theoretically, more effective and less vulnerable to resistance. Hence, this rationale may also contribute to design inhibitors with a higher resilience to resistance since multiple mutations (in sites which have shown, *a priori*, high degree of conservation) would be required for the virus become resistant.

## 3 Conclusion

This study discloses a promising anti-CoV strategy directed to highly conserved druggable S regions, resulting from a comprehensive large-scale sequence analysis and structural characterization of protein domains across SARSr- and MERSr-CoVs.

The most promising conserved druggable regions highlighted in this study consisted of the RBD and SD1 (in the S1 subunit) and the FP, CR, HR1 and CH (in the S2 subunit) of S protein. Ultimately, the identification of CDR and conserved druggable sites/domains depends on the range of the anti-CoV strategy. A strategy which only targets the hSARSr-CoVs includes additional CDR within the RBD and FP domain, in addition to the CDR described for the RBD and SD1 (S1 subunit) of the SARSr- and MERSr-CoVs. Priority should be given to the RBD and SD1 (in S1) and to the CH (in S2), which exhibited a higher antiviral target potential among all protein conformations from all Beta-CoVs included in this study.

We have identified 181 new potential hot spot residues for hSARSr-CoVs and 72 new hot spot residues for SARSr- and MERSr-CoVs, which have not been described before in the literature. These hotspots are mostly surface exposed and represent attractive targets for molecular or pharmacological modulation. We hypothesize that an anti-CoV strategy targeting potential functionally or structurally highly conserved sites, provides a higher resilience to resistance development and can be potentially useful against the new SARS-CoV-2 and a broad spectrum of Beta-CoVs.

Further structural studies regarding the conservation and druggability of CoV proteins should also be extended to the RNA-dependent RNA-polymerase or to the main proteinase, considering its important function during the virus replication cycle. The strategies used in our study have been previously applied with success to structural and nonstructural proteins from other respiratory virus, and can be broadened applied to other re-emerging viral pathogens such as Zika virus, Ebola virus, as well as re-emerging CoV for a structure-based prediction of druggable biologic targets.

Ultimately, this study lays the foundation for successful structure-based design and discovery of chemical inhibitors, antibodies or other therapeutic modalities that target the S protein of Beta-CoVs.

## 4 Material and methods

### 4.1 Dataset construction and sequence analysis

All nucleotide sequences with a complete coding region of worldwide circulating human beta-CoVs including SARS-CoV-2, SARS-CoVs and MERS-CoVs, along with bat-SL-CoVs were retrieved on 24 March 2020 from sequence databases. These included: GISAID's EpiCov™ database (www.gisaid.org) [77], [78], GenBank (https://www.ncbi.nlm.nih.gov/genbank/sars-cov-2-seqs/) [79], 2019 Novel Coronavirus Resource (2019nCoVR) (https://bigd.big.ac.cn/ncov) [80], NIAID Virus Pathogen Resource (ViPR) database (https://www.viprbrc.org) [81] and NCBI Virus database (https://www.ncbi.nlm.nih.gov/labs/virus) [82].

The S surface glycoprotein encoded by the S gene was selected for study. The S gene was divided into the S1 and S2 subunits according to the S1/S2 cleavage site. The nucleotide sequence alignment was performed using Multiple Alignment Fast Fourier Transform (MAFFT) available at https://www.ebi.ac.uk/Tools/msa/mafft/ [83], [84]. The aa numbering was established according to the consensus sequence of the SARS-CoV-2 sequence alignment.

A total of 1,065 CoV S1 sequences and 1,096 CoV S2 sequences were included. Particularly, 674 SARS-CoV-2 isolates, 114 SARS-CoV isolates, 50 Bat-SL-CoV isolates and 248 MERS-CoV isolates for the S1 subunit, and 682 SARS-CoV-2 isolates, 116 SARS-CoV isolates, 50 Bat-SL-CoV isolates and 248 MERS-CoV isolates for the S2 subunit (depicted in Table 1).

All continents are represented with strains from the SARS-CoV-2. Specifically, Asian countries (n = 230 isolates) included Cambodia, China, Georgia, Hong Kong, India, Japan, Kuwait, Malaysia, Nepal, Saudi Arabia, Singapore, South Korea, Taiwan, Thailand and Vietnam. African countries (n = 3 isolates) included Democratic Republic of the Congo, Nigeria and South Africa. The North, Central and South America are represented by Canada, United States of America, Mexico, Panama, Brazil and Chile (n = 129 isolates). Twenty European countries were included in the study, namely Belgium, Czech Republic, Denmark, England, Finland, France, Germany, Hungary, Ireland, Italy, Lithuania, Luxemburg, Netherlands, Poland, Portugal, Scotland, Spain, Sweden, Switzerland and Wales (n = 298 isolates). Strains from Australia and New Zealand (Oceania) were also included (n = 26 isolates).

Multiple sequence alignment (MSA) visualization and the study of protein primary structure and length variation distributions among each beta-CoVs, was performed using MEGA X (https://www.megasoftware.net/) [85].

### 4.2 Conservation analysis

The aa conservation in each position was calculated using the Valdar scoring method from the Jalview AACons Web server (v2.11) [86], [87]. The weighted scores incorporated into this conservation method consider the sequence redundancy in the MSA. This method enables the normalization against redundancy and bias in the MSA (reducing both the effect of bias sampling and penalizing gaps) with the benefit of no loss of evolutionary information [86]. This scoring system has been previously applied with success [88], [89], [90]. The conservation degree is presented as a numerical score, within a range of 0 (highly variable site) to 11 (highly conserved site), for each aa in the protein sequence alignment [86], [87]. Residues with conservation score of 11 correspond to absolute conserved sites (100*%* aa identity). Residues were considered highly conserved for conservation score values ≥ 10, conserved for conservation score of 7–9 and variable for conservation score values < 7 [90], [91].

The SARS-CoV-2 was used as a reference for the alignment consensus (Jalview v 2.11.0 software). The conservation scores were calculated for each aa position within the S protein for three different groups: (a) SARS-CoV-2; (b) hSARSr-CoV; and (c) SARSr- and MERSr-CoV. A group comparing hSARS-CoVs and Bat-SL-CoVs was also briefly studied. The conservation scores for the (b) hSARSr-CoVs and the (c) SARSr- and MERSr-CoVs were mapped onto the S monomeric structure (SARS-CoV-2, PDB entry 6VYB).

### 4.3 Druggability analysis

Druggability studies were performed using the newly available SARS-CoV-2 crystallographic structures. Crystallographic structures of SARS-CoV-2 S protein trimer (PDB entries 6VSB, 6VXX, 6VYB) [6], [92] and RBD (PDB entries 6VW1, 6LZG, 6M0J) [12], [28], [44] were selected for study and retrieved from the RCSB Protein Data Bank (www.rcsb.org) [93]. Each structure was accurately prepared by removing coordinated molecules and bounded ligands, protonation and energy minimization using MOE 2015.10001 program [94]. To date, the available S crystal structures are not resolved for the signal peptide (S1 subunit), heptad repeat 2 (HR2), transmembrane region and CP (S2 subunit), which limit the druggability prediction and further analysis of these regions.

The druggable sites/residues within the selected structures were predicted from a consensus of a triple strategy, following the same rational described by Trigueiro-Louro et al. 2019, using the webservers DGSS at https://proteins.plus, the commercial software MOE-SiteFinder (SF) from Chemical Computing Group and (when applied) the PockDrug-Server (PDS) at http://pockdrug.rpbs.univ-paris-diderot.fr/ [90], [94], [95], [96], [97].

The DGSS server is based on a grid-based method incorporated in a support vector machine model for druggability predictions. The algorithm is based on geometric and physicochemical descriptors and provides a raw druggability score for each pocket, ranging from 0 (undruggable) to 1 (druggable). Pockets with a DGSS druggability score ≥ 0.4 were considered druggable [96]. The druggability threshold was previously defined by Volkamer and the methodology was applied with success by other authors [90], [96], [98], [99], [100]*.*

A triple consensus-based strategy was applied since some variation may be observed among different druggability prediction methods [90], [97]. Considering the PockDrug-Server is able to predict the pocket(s) druggability for a maximum file size of 1 MB, only the crystallographic structures of the SARS-Cov-2 S RBD were studied using this webserver.

The druggability prediction for the S monomer and trimer structures (PDB IDs: 6VSB, 6VXX, 6VYB) was studied for each individual structure using the bioinformatics tools: (a) SF and (b) DGSS and according to the number of structures ascribed to each group. The druggability analysis for each individual SARS-CoV-2 S RBD structure (PDB entries 6VW1, 6LZG, 6M0J) was performed with the three bioninformatic tools: (a) SF, (b) DGSS and (c) PDS. Subsequently, a comparative analysis was performed by merging the druggability information from the three bioinformatic tools for each group: (1) S monomer structures; (2) S RBD structures; and (3) S trimer structures.

The last stage of the study consisted of a global comparative analysis of the most prevailing druggable sites identified by the bioinformatics tools and shared by all the structures within each group. The druggability consensus of each group was manually analysed in parallel with the conservation data to identify druggable binding sites/pockets or potential hot spots residues that overlap to the conserved regions/residues of the CoV S proteins.

The coordinates of the highly score CDPs were mapped onto the three-dimensional S trimer structure using PyMOL Molecular Graphics System, version 1.8 (Schrödinger, LLC; https://pymol.org) [101]. This rational has been previously applied with success in studies performed by the same authors and other studies regarding the druggability analysis of influenza virus proteins [90], [102], [103].

## CRediT authorship contribution statement

**João Trigueiro-Louro:** Conceptualization, Methodology, Software, Investigation, Writing - original draft, Writing - review & editing, Supervision. **Vanessa Correia:** Methodology, Software, Investigation, Writing - original draft, Writing - review & editing. **Inês Figueiredo-Nunes:** Methodology, Software. **Marta Gíria:** Investigation, Writing - original draft, Writing - review & editing. **Helena Rebelo-de-Andrade:** Conceptualization, Methodology, Investigation, Writing - review & editing, Supervision, Project administration, Funding acquisition.

## Declaration of Competing Interest

The authors declare that they have no known competing financial interests or personal relationships that could have appeared to influence the work reported in this paper.

## Appendix A Supplementary data

Supplementary figure 1 **Representation of the conservation-score distribution of the Spike S1 and S2 residues.** The cs were calculated for each aa position within the S protein for three different groups: (a) SARS-CoV-2; (b) hSARSr-CoV; and (c) SARSr and MERSr-CoV. The white numbers (below each aa position) represent the cs of each individual aa, on a scale varying from 0 (most variable) to 11 (most conserved); a score of 11 indicates 100% identity across the considered beta-CoVs. The regions composed of ≥ 10 continuous conserved aa (score >7) are displayed above the conservation-score bars.

Supplementary figure 2 **Comparison of the druggability prediction for the Spike monomer open and closed state conformations.** Druggability prediction based on estimation by the pocket bioinformatic tools – DGSS and SF – for the open (PDB IDs: 6VYB and 6VSB) and closed (PDB IDs: 6VXX and 6VYB) S monomer structures. The potential druggable sites/residues were coloured in light blue and dark blue, following the score scale (0-4) depicted in the figure. Only sites/residues graded with druggability score ≥3 for one conformation group and graded with a score <3 for the opposite conformation have been highlighted. The additional druggable sites/residues found for each conformation group are highlighted with a blue rectangle.

Supplementary figure 3 **Overall alignment of Spike monomer druggability along with the conservation scores for each residue position.** The druggability prediction was based on the descriptors algorithm of each pocket bioinformatics tool: SF and DGSS. The potential conserved druggable sites/residues are marked with an asterisk and the top-ranked hot spots are marked with a target. The conserved druggable pockets allocated to each site/residue along with the secondary structure elements are indicated at the top of the picture.

Supplementary table 1 **Druggability prediction for the Spike monomer conformation.** Potential druggable pockets of S monomer conformers (closed and open) along with the respective aa residues predicted by DGSS. Only pockets with a druggability score ≥ 0.4 and with ≥10 aa were considered.

Supplementary table 2 **Consensus druggable pockets predicted for the Spike crystallographic structures (RBD, monomer and trimer) of SARS-CoV-2.** All protein conformations (open, semi-open and closed conformation, when applied) were considered for the S monomer (CDP.M) and S trimer (CDTP.T). The aa composition of each CDP and the corresponding location (S subunit and domain) are described in the table.

Supplementary table 3 **Top-ranked hot spots for drug targeting among beta-CoVs.** The T-RHS for each group (1) S monomer (2) S-RBD and (3) S trimer were predicted based on the descriptors algorithm from all the pocket bioinformatics tools: SF, DGSS and PDS, with respected to the hSARSr-CoVs and the SARSr- and MERSr-CoVs.

Supplementary table 4 **Druggability prediction for the Spike-RBD.** Potential druggable pockets of S-RBD along with the respective aa residues predicted by DGSS. Only pockets with a druggability score ≥ 0.4 and with ≥ 10 aa were considered.

Supplementary table 5 **Druggability prediction for the Spike trimer conformation.** Potential druggable pockets of S trimer conformers (closed, semi-open and open) along with the respective aa residues predicted by DGSS. Only pockets with a druggability score ≥ 0.4 and with ≥ 10 aa were considered.

Supplementary table 6 **Conserved druggable sites/residues for drug targeting shared by the Spike monomer and trimer structures.** The CDR shared by both groups (S monomer and trimer) were predicted based on the descriptors algorithm of the pocket bioinformatics tools: SF and DGSS, with respected to the hSARSr-CoVs and the SARSr- and MERSr-CoVs.

Supplementary table 7 **New potential hot spots residues for drug targeting.** Hot spot residues identified in this study which, to the best of our knowledge, have not been described before in the literature.
