# Supplementary material for: Unlocking COVID therapeutic targets: A structure-based rationale against SARS-CoV-2, SARS-CoV and MERS-CoV Spike
Source: Comput Struct Biotechnol J. 2020 Jul 31;18:2117–31. doi: 10.1016/j.csbj.2020.07.017 (PMC7452956; doi:10.1016/j.csbj.2020.07.017)
Supplement: Supplementary figure 1 — Representation of the conservation-score distribution of the Spike S1 and S2 residues. The cs were calculated for each aa position within the S protein for three different groups: (a) SARS-CoV-2; (b) hSARSr-CoV; and (c) SARSr and MERSr-CoV. The white numbers (below each aa position) represent the cs of each individual aa, on a scale varying from 0 (most variable) to 11 (most conserved); a score of 11 indicates 100% identity across the considered beta-CoVs. The regions composed of ≥ 10 continuous conserved aa (score >7) are displayed above the conservation-score bars. [file mmc1.pdf]

(a) SARS-CoV-2

S1

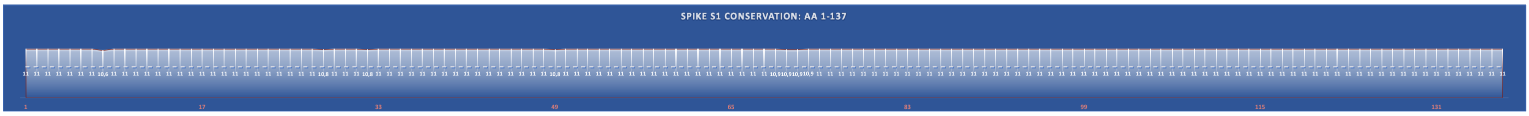

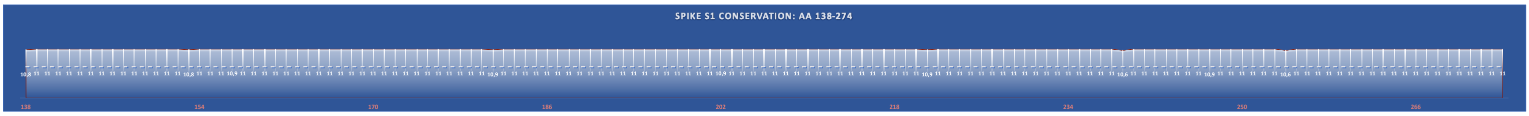

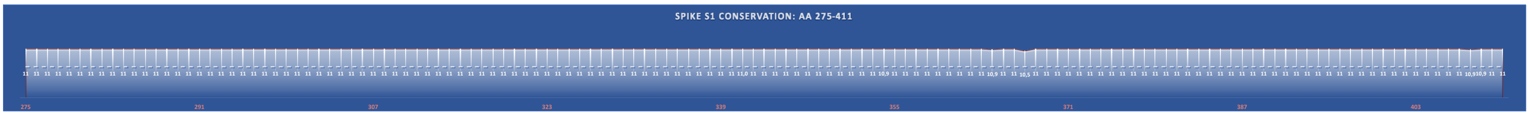

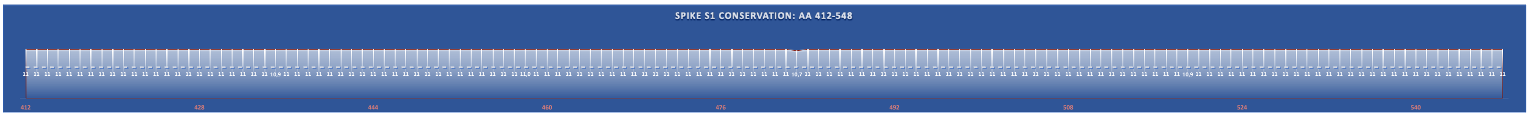

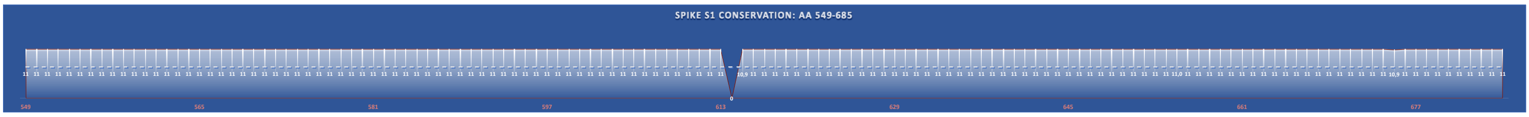

S2

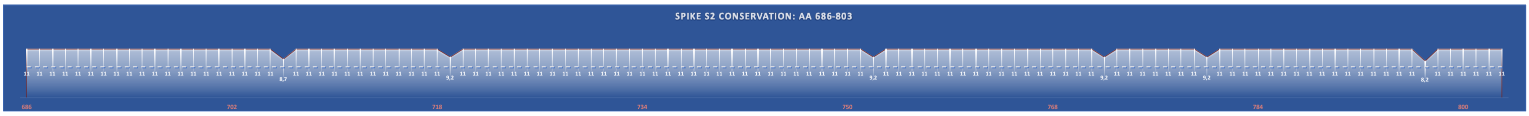

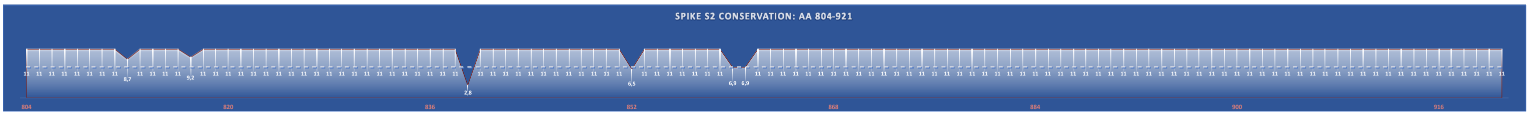

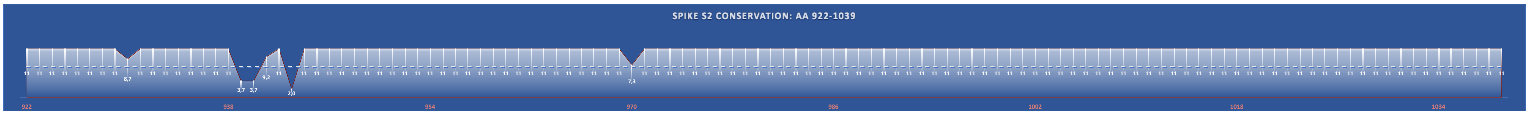

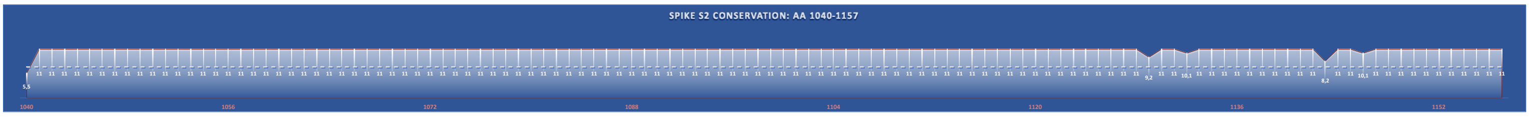

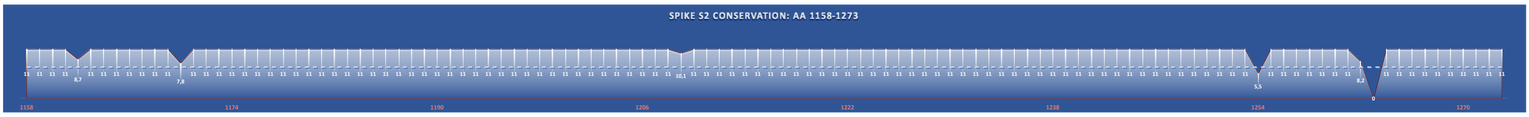

(b) human SARSr-CoV

S1

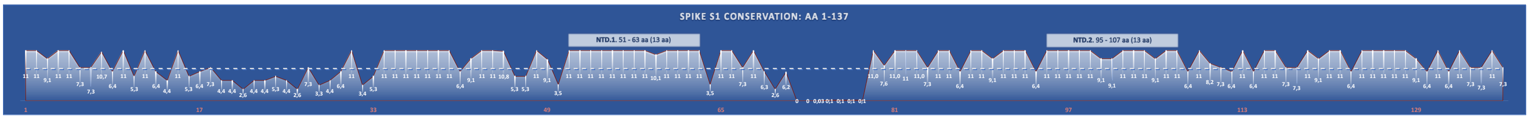

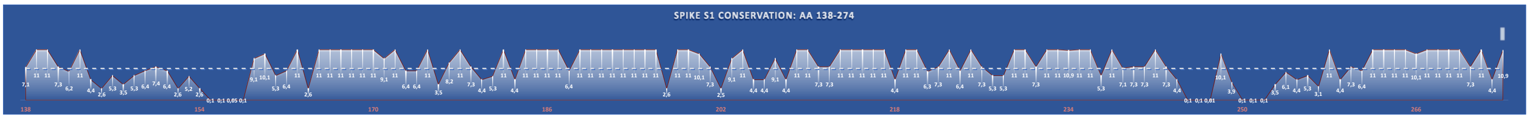

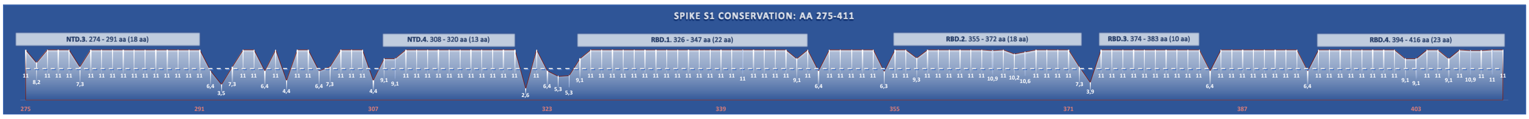

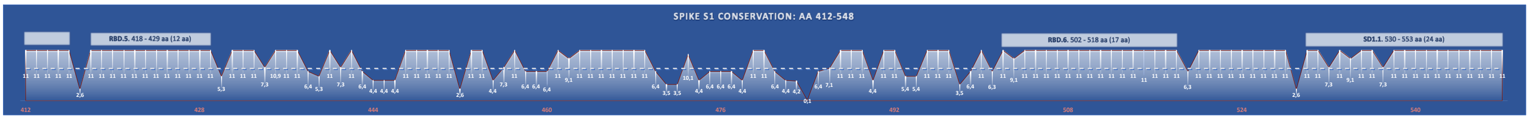

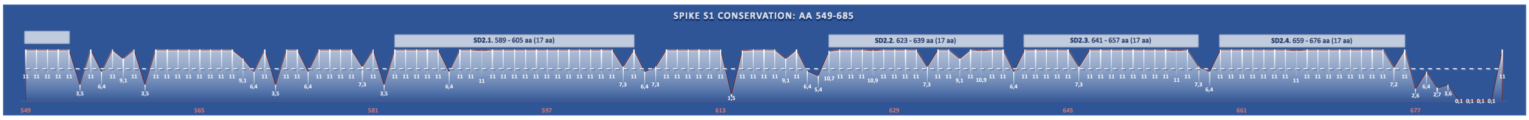

S2

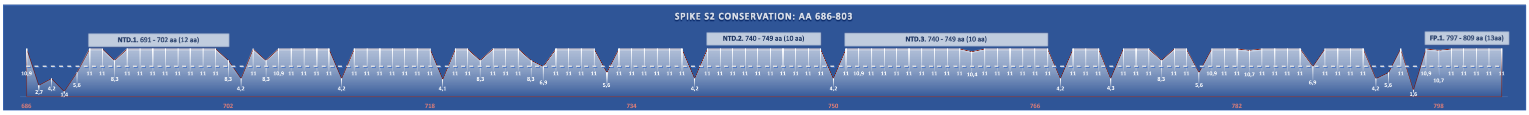

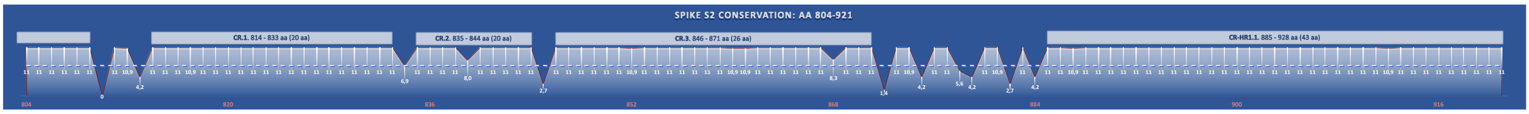

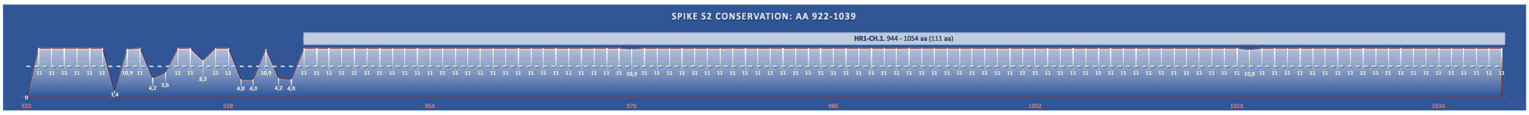

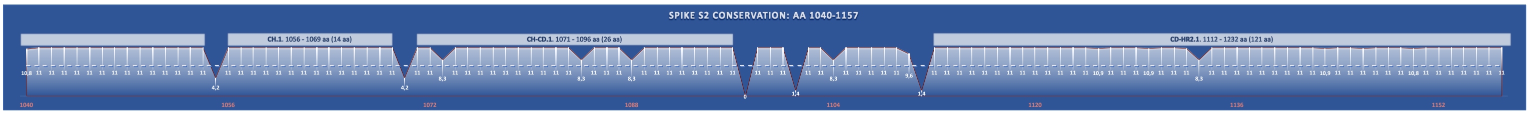

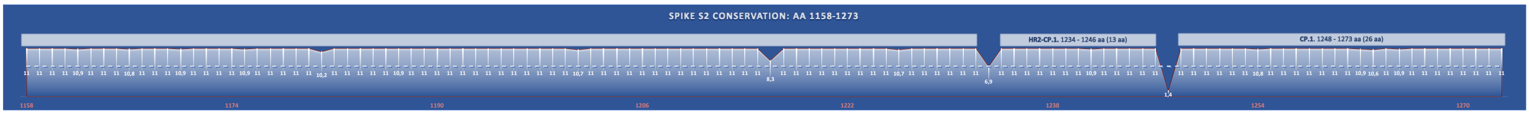

(c) SARSr and MERSr-CoV

S1

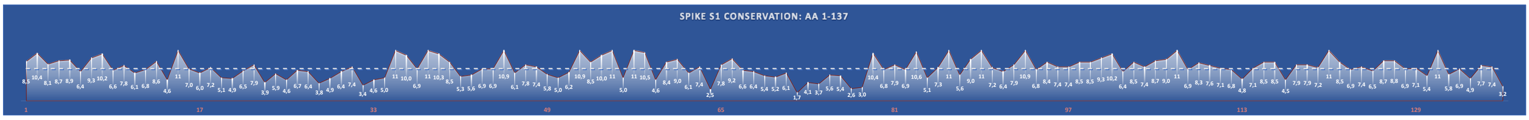

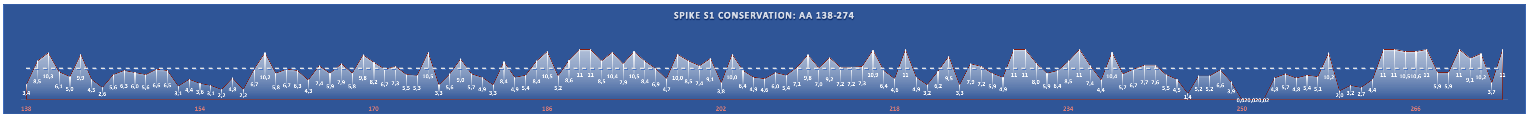

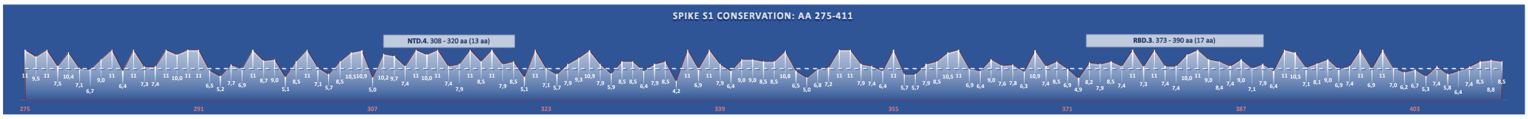

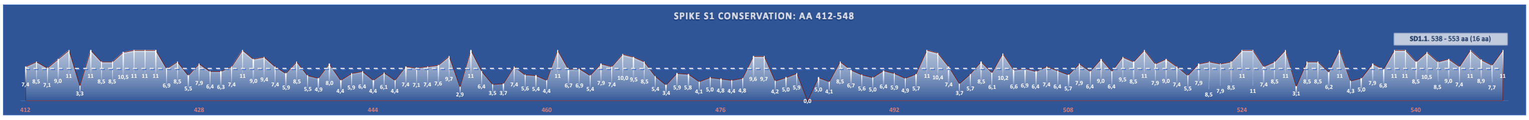

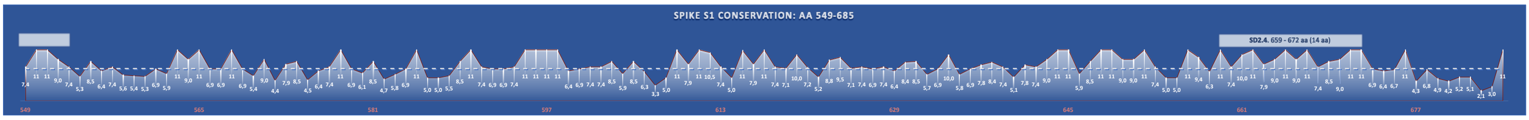

S2

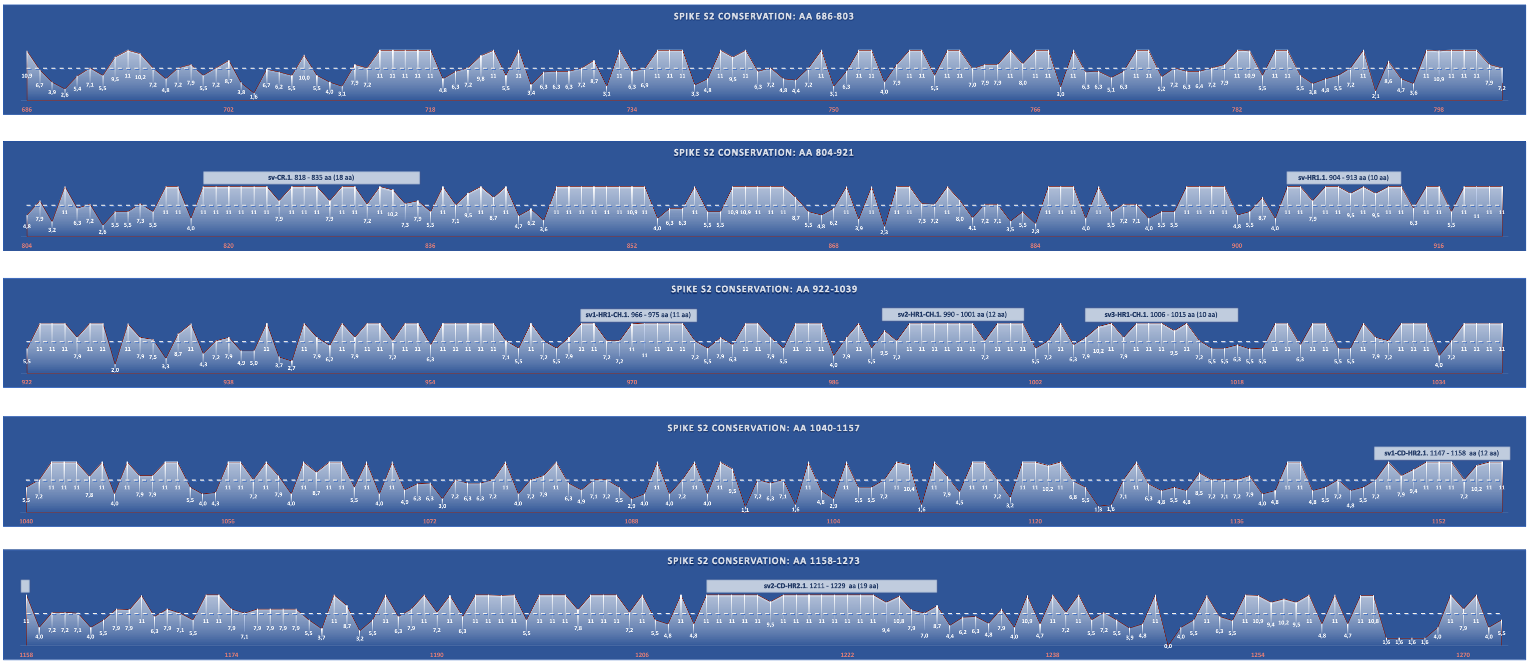

Figure S-1. Representation of the conservation-score distribution of the Spike S1 and S2 residues. The conservation scores (cs) were calculated for each aa position within the S protein for three different groups: **(a)** SARS-CoV-2; **(b)** human SARSr-CoV; and **(c)** SARSr and MERSr-CoV**.** The white numbers (below each aa position) represent the cs of each individual aa, on a scale varying from 0 (most variable) to 11 (most conserved); a score of 11 indicates 100% identity across the considered beta-CoVs. In (b) and (c) the regions composed of ≥ 10 continuous conserved aa (score >7) are displayed above the conservation-score bars.
