# Supplementary figures and images for: Unlocking COVID therapeutic targets: A structure-based rationale against SARS-CoV-2, SARS-CoV and MERS-CoV Spike
Source: Comput Struct Biotechnol J. 2020 Jul 31;18:2117–31. doi: 10.1016/j.csbj.2020.07.017 (PMC7452956; doi:10.1016/j.csbj.2020.07.017)

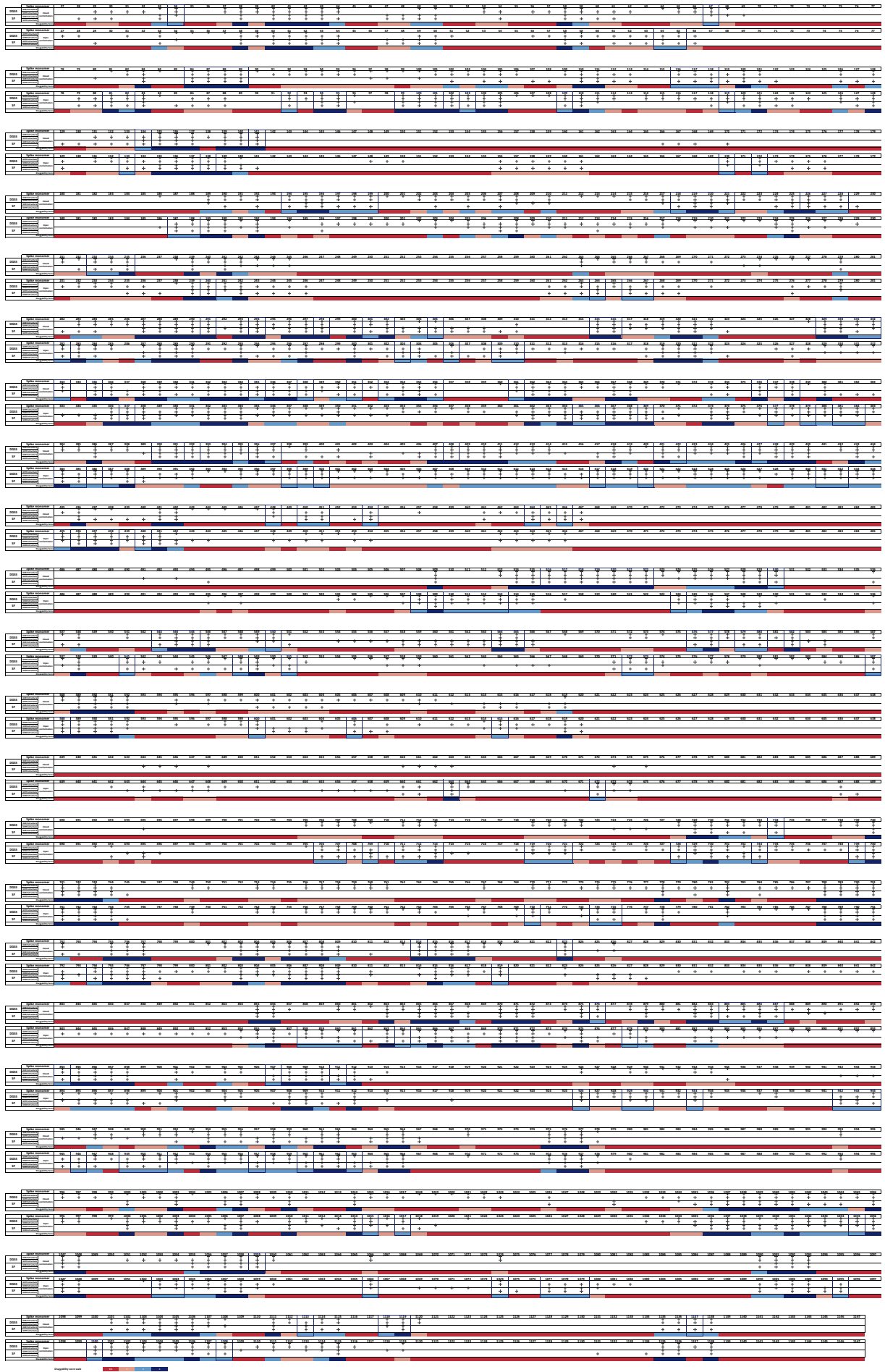

Supplement: Supplementary figure 2 — Comparison of the druggability prediction for the Spike monomer open and closed state conformations. Druggability prediction based on estimation by the pocket bioinformatic tools – DGSS and SF – for the open (PDB IDs: 6VYB and 6VSB) and closed (PDB IDs: 6VXX and 6VYB) S monomer structures. The potential druggable sites/residues were coloured in light blue and dark blue, following the score scale (0-4) depicted in the figure. Only sites/residues graded with druggability score ≥3 for one conformation group and graded with a score <3 for the opposite conformation have been highlighted. The additional druggable sites/residues found for each conformation group are highlighted with a blue rectangle. [file mmc2.pdf]

### S1 SUBUNIT

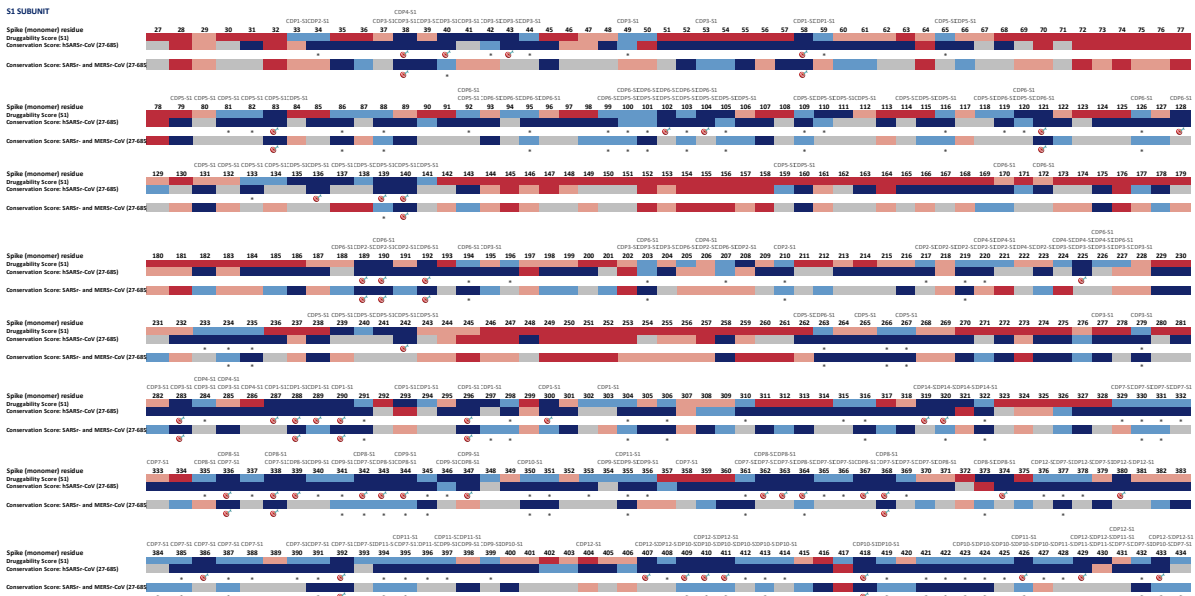

## S2 SUBUNIT

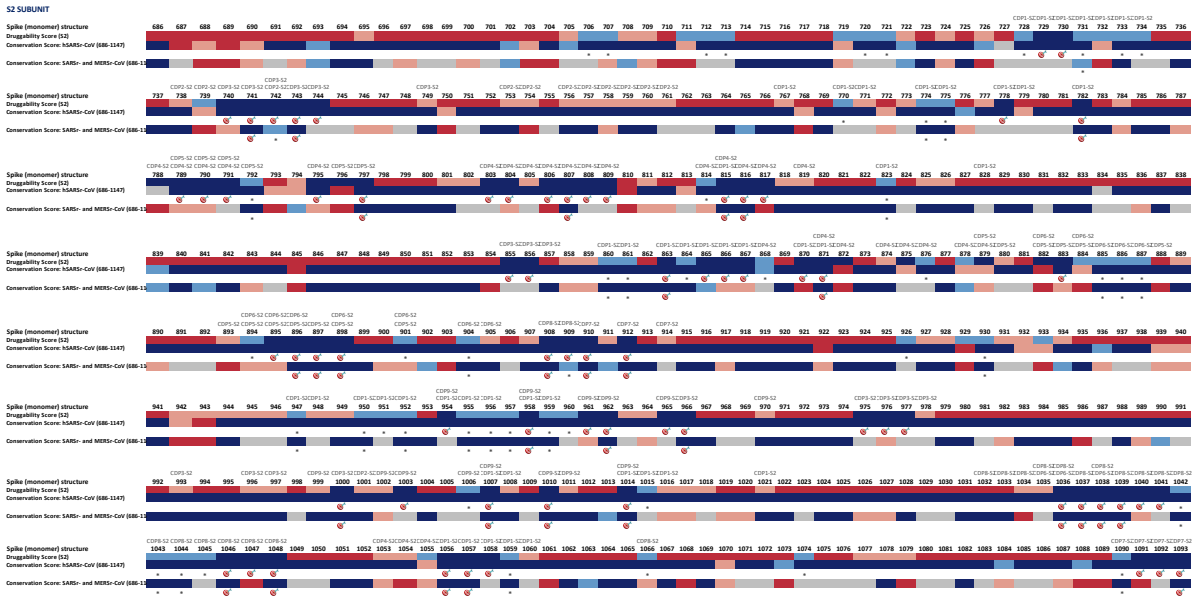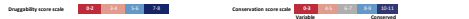

Supplement: Supplementary figure 3 — Overall alignment of Spike monomer druggability along with the conservation scores for each residue position. The druggability prediction was based on the descriptors algorithm of each pocket bioinformatics tool: SF and DGSS. The potential conserved druggable sites/residues are marked with an asterisk and the top-ranked hot spots are marked with a target. The conserved druggable pockets allocated to each site/residue along with the secondary structure elements are indicated at the top of the picture. [file mmc3.pdf]
