# Supplementary material for: Unlocking COVID therapeutic targets: A structure-based rationale against SARS-CoV-2, SARS-CoV and MERS-CoV Spike
Source: Comput Struct Biotechnol J. 2020 Jul 31;18:2117–31. doi: 10.1016/j.csbj.2020.07.017 (PMC7452956; doi:10.1016/j.csbj.2020.07.017)
Supplement: Supplementary table 2 — Consensus druggable pockets predicted for the Spike crystallographic structures (RBD, monomer and trimer) of SARS-CoV-2. All protein conformations (open, semi-open and closed conformation, when applied) were considered for the S monomer (CDP.M) and S trimer (CDTP.T). The aa composition of each CDP and the corresponding location (S subunit and domain) are described in the table. [file mmc5.docx]

**Table S-2. Consensus druggable pockets predicted for the Spike crystallographic structures (RBD, monomer and trimer) of SARS-CoV-2.** All protein conformations (open, semi-open and closed conformation, when applied) were considered for the S monomer (CDP.M) and S trimer (CDTP.T). The aa composition of each CDP and the corresponding location (S subunit and domain) are described in the table.

| **Spike Structure** | **Subunit** | **Domain** | **CDP** | **Size** | **Residues** |
| --- | --- | --- | --- | --- | --- |
| **RBD** |  | RBD | CDP1 | 16 aa | 335, 336, 338, 339, 342, 343, 344, 364, 367, 368, 371, 373, 374, 436, 441, 509 |
|  |  | RBD | CDP2 | 15 aa | 365, 366, 368, 369, 377, 379, 384, 387, 388, 432, 434, 513, 515, 524, 525 |
|  |  | RBD | CDP3 | 14 aa | 454, 456, 457, 458, 467, 469, 471, 472, 473, 474, 480, 481, 482, 491 |
|  |  | RBD | CDP4 | 11 aa | 409, 410, 411, 412, 413, 414, 418, 419, 423, 424, 425 |
|  |  | RBD | CDP5 | 11 aa | 355, 396, 426, 428, 429, 430, 431, 464, 514, 515, 516 |
|  |  | RBD | CDP6 | 12 aa | 340, 341, 344, 346, 347, 348, 354, 355, 356, 397, 398, 399 |
|  |  | RBD | SP1 | 8 aa | 376, 378, 380, 407, 408, 410, 411, 433 |
|  |  | RBD | SP2 | 8 aa | 403, 453, 494, 495, 496, 497, 501, 505 |
|  |  |  |  |  |  |
| **Spike Monomer** | S1 | NTD | CDP1M-S1 | 14 aa | 33, 58, 59, 287, 288, 289, 290, 293, 294, 296, 297, 300, 306, 606 |
|  | S1 | NTD | CDP2M-S1 | 13 aa | 34, 189, 190, 191, 206, 208, 210, 217, 218, 219, 220, 221, 223 |
|  | S1 | NTD | CDP3M-S1 | 25 aa | 37, 38, 39, 40, 41, 42, 43, 44, 49, 53, 195, 202, 203, 204, 224, 225, 226, 227, 228, 277, 279, 282, 283, 284, 285 |
|  | S1 | NTD | CDP4M-S1 | 11 aa | 38, 206, 220, 221, 222, 224, 225, 226, 284, 285, 286 |
|  | S1 | NTD | CDP5M-S1 | 47 aa | 65, 66, 79, 80, 81, 82, 83, 84, 92, 100, 101, 102, 103, 104, 105, 106, 109, 110, 111, 112, 114, 115, 116, 118, 120, 131, 132, 133, 134, 135, 136, 137, 138, 139, 140, 141, 159, 160, 238, 239, 240, 241, 242, 243, 262, 265, 267 |
|  | S1 | NTD | CDP6M-S1 | 28 aa | 92, 93, 94, 95, 96, 99, 100, 101, 102, 103, 104, 119, 120, 121, 126, 128, 170, 172, 188, 190, 192, 194, 203, 205, 207, 226, 227, 263 |
|  | S1 | RBD | CDP7M-S1 | 61 aa | 329, 330, 331, 332, 333, 336, 338, 342, 358, 361, 362, 363, 365, 366, 368, 369, 370, 377, 379, 384, 385, 386, 387, 388, 390, 391, 392, 393, 395, 431, 432, 434, 513, 515, 516, 517, 518, 519, 520, 521, 522, 523, 524, 525, 526, 527, 528, 529, 530, 543, 544, 545, 546, 564, 565, 576, 577, 578, 579, 580, 582 |
|  | S1 | RBD | CDP8M-S1 | 22 aa | 335, 336, 338, 339, 342, 343, 344, 347, 362, 363, 364, 367, 368, 371, 373, 374, 436, 437, 438, 441, 442, 509 |
|  | S1 | RBD | CDP9M-S1 | 12 aa | 340, 341, 344, 346, 347, 354, 355, 356, 397, 398, 399, 511 |
|  | S1 | RBD | CDP10M-S1 | 18 aa | 350, 400, 409, 410, 411, 412, 413, 414, 418, 419, 423, 424, 425, 426, 427, 433, 463, 512 |
|  | S1 | RBD | CDP11M-S1 | 17 aa | 355, 394, 395, 396, 397, 398, 426, 428, 429, 430, 431, 464, 512, 513, 514, 515, 516 |
|  | S1 | RBD | CDP12M-S1 | 19 aa | 376, 378, 380, 381, 404, 407, 408, 410, 411, 429, 430, 431, 433, 434, 435, 436, 503, 508, 510 |
|  | S1 | SD1 | CDP13M-S1 | 12 aa | 541, 546, 547, 548, 549, 550, 572, 573, 574, 587, 588, 589 |
|  | S1 | SD1+SD2 +NTD | CDP14M-S1 | 14 aa | 319, 320, 321, 322, 536, 537, 538, 549, 551, 589, 590, 591, 592, 619 |
|  | S2 | NTD+CR+HR1+CH | CDP1M-S2 | 48 aa | 728, 729, 730, 731, 732, 733, 734, 767, 770, 771, 774, 775, 778, 779, 782, 815, 823, 828, 860, 861, 863, 864, 865, 866, 867, 870, 871, 947, 948, 950, 951, 952, 954, 955, 956, 958, 959, 1007, 1008, 1014, 1015, 1016, 1021, 1056, 1057, 1058, 1059, 1060 |
|  | S2 | NTD | CDP2M-S2 | 12 aa | 738, 739, 742, 753, 754, 756, 757, 758, 759, 760, 761, 1001 |
|  | S2 | NTD+CR+HR1+CH | CDP3M-S2 | 18 aa | 740, 741, 742, 743, 744, 749, 855, 856, 857, 966, 975, 976, 977, 978, 993, 996, 997, 1000 |
|  | S2 | FP+CR+CH | CDP4M-S2 | 28 aa | 788, 789, 790, 791, 795, 803, 804, 805, 806, 807, 808, 809, 814, 815, 816, 817, 819, 868, 871, 872, 874, 875, 876, 878, 879, 1053, 1054, 1055 |
|  | S2 | FP+CR | CDP5M-S2 | 19 aa | 789, 790, 791, 792, 796, 797, 879, 880, 882, 883, 884, 888, 893, 894, 895, 896, 897, 898, 901 |
|  | S2 | CR+CH | CDP6M-S2 | 15 aa | 882, 884, 885, 886, 887, 894, 895, 896, 898, 901, 904, 905, 1035, 1036, 1038 |
|  | S2 | HR1+CD | CDP7M-S2 | 18 aa | 910, 912, 914, 1090, 1091, 1092, 1093, 1104, 1105, 1106, 1107, 1108, 1111, 1112, 1113, 1118, 1119, 1120 |
|  | S2 | CR+CH | CDP8M-S2 | 20 aa | 908, 909, 1032, 1033, 1034, 1035, 1036, 1037, 1038, 1039, 1040, 1041, 1042, 1043, 1044, 1045, 1046, 1047, 1048, 1066 |
|  | S2 | HR1+CH | CDP9M-S2 | 14 aa | 954, 958, 961, 962, 965, 970, 999, 1002, 1003, 1006, 1007, 1010, 1011, 1014 |
|  |  |  |  |  |  |
| **Spike Trimer** | S1 | NTD | CDP1T-S1 | 13 aa | 33, 58, 59, 287, 288, 289, 290, 293, 294, 296, 297, 300, 306 |
|  | S1 | NTD | CDP2T-S1 | 13 aa | 34, 91, 189, 190, 191, 206, 208, 210, 217, 219, 221, 222, 223 |
|  | S1 | NTD+RBD | CDP3T-S1 | 42 aa | Chain1\| 38, 39, 40, 41, 42, 43, 44, 49, 200, 202, 203, 204, 225, 226, 227, 228, 230, 277, 279, 282, 283, 284, 285. Chain2\| 355, 357, 393, 394, 395, 396, 426, 428, 429, 430, 464, 514, 515, 516, 518, 519, 520, 521, 523 |
|  | S1 | NTD | CDP4T-S1 | 30 aa | 65, 82, 84, 86, 90, 91, 92, 100, 101, 102, 103, 104, 105, 106, 118, 120, 134, 135, 136, 139, 140, 141, 238, 239, 240, 241, 242, 243, 265, 267 |
|  | S1 | NTD+RBD | CDP5T-S1 | 30 aa | Chain1\| 86, 195, 196, 197, 198, 199, 200, 229, 230, 231, 232, 233, 234, 235. Chain2\| 353, 355, 395, 396, 426, 428, 429, 430, 462, 463, 464, 465, 466, 514, 515, 516 |
|  | S1 | NTD | CDP6T-S1 | 20 aa | 92, 94, 99, 101, 102, 103, 104, 119, 120, 121, 126, 128, 190, 192, 194, 203, 205, 207, 226, 227 |
|  | S1 | RBD | CDP7T-S1 | 16 aa | 335, 336, 338, 339, 342, 343, 362, 363, 364, 365, 367, 368, 371, 373, 374, 436 |
|  | S1 | RBD | CDP8T-S1 | 13 aa | 340, 341, 344, 346, 347, 348, 354, 355, 356, 397, 398, 399, 511 |
|  | S1 | RBD | CDP9T-S1 | 25 aa | 349, 350, 400, 401, 402, 403, 406, 413, 414, 415, 417, 418, 421, 422, 443, 448, 451, 452, 453, 454, 495, 497, 507, 508, 509 |
|  | S1 | RBD | CDP10T-S1 | 13 aa | 409, 410, 411, 412, 413, 414, 418, 419, 423, 424, 425, 426, 427 |
|  | S1 | RBD | CDP11T-S1 | 18 aa | 350, 351, 352, 353, 418, 419, 420, 421, 422, 423, 424, 454, 462, 463, 464, 465, 466, 467 |
|  | S1 | NTD+SD1+SD2 | CDP12T-S1 | 23aa | 317, 319, 320, 321, 322, 536, 537, 538, 549, 551, 557, 566, 567, 568, 569, 570, 571, 574, 589, 590, 591, 592, 619 |
|  | S1 | NTD+SD1 | SP1T-S1 | 9 aa | 38, 206, 220, 221, 222, 224, 284, 285, 560 |
|  | S1 | RBD | SP2T-S1 | 8 aa | 376, 378, 380, 407, 408, 410, 411, 433 |
|  | S2 | NTD+CR+HR1+CH | CDP1T-S2 | 46 aa | 728, 729, 730, 731, 732, 733, 734, 767, 770, 771, 774, 775, 777, 778, 781, 782, 815, 823, 860, 861, 862, 863, 864, 865, 866, 867, 870, 947, 950, 951, 954, 955, 958, 959, 1007, 1008, 1014, 1015, 1016, 1021, 1055, 1056, 1057, 1058, 1059, 1060 |
|  | S2 | NTD+HR1+CH | CDP2T-S2 | 162 aa | Chain1\| 728, 731, 735, 736, 756, 758, 759, 762, 763, 765, 766, 769, 770, 771, 773, 774, 776, 777, 947, 950, 951, 954, 957, 958, 961, 962, 965, 970, 994, 995, 998, 999, 1001, 1002, 1003, 1004, 1005, 1006, 1007, 1008, 1009, 1010, 1011, 1012, 1013, 1014, 1015, 1016, 1017, 1018, 1019, 1020, 1021, 1022. Chain2\| 728, 731, 735, 736, 756, 758, 759, 762, 763, 765, 766, 769, 770, 771, 773, 774, 776, 777, 947, 950, 951, 954, 957, 958, 961, 962, 965, 970, 994, 995, 998, 999, 1001, 1002, 1003, 1004, 1005, 1006, 1007, 1008, 1009, 1010, 1011, 1012, 1013, 1014, 1015, 1016, 1017, 1018, 1019, 1020, 1021, 1022. Chain 3\| 728, 731, 735, 736, 756, 758, 759, 762, 763, 765, 766, 769, 770, 771, 773, 774, 776, 777, 947, 950, 951, 954, 957, 958, 961, 962, 965, 970, 994, 995, 998, 999, 1001, 1002, 1003, 1004, 1005, 1006, 1007, 1008, 1009, 1010, 1011, 1012, 1013, 1014, 1015, 1016, 1017, 1018, 1019, 1020, 1021, 1022 |
|  | S2 | NTD+FP+CR+CH | CDP3T-S2 | 26 aa | 702, 788, 789, 790, 791, 795, 805, 806, 807, 809, 814, 815, 816, 819, 868, 871, 872, 874, 875, 878, 879, 882, 1052, 1053, 1054, 1055 |
|  | S2 | NTD+CR+CH+CD | CDP4T-S2 | 81 aa | Chain1\| 712, 713, 714, 715, 908, 909, 1035, 1039, 1040, 1041, 1042, 1043, 1044, 1045, 1046, 1047, 1048, 1049, 1066, 1067, 1069, 1070, 1071, 1072, 1094, 1095, 1096. Chain2\| 712, 713, 714, 715, 908, 909, 1035, 1039, 1040, 1041, 1042, 1043, 1044, 1045, 1046, 1047, 1048, 1049, 1066, 1067, 1069, 1070, 1071, 1072, 1094, 1095, 1096 Chain3\| 884, 885, 886, 887, 889, 890, 891, 892, 894, 895, 896, 900, 901, 904, 905, 908, 909, 1031, 1032, 1033, 1035, 1036, 1037, 1038, 1039, 1040, 1041. |
|  | S2 | NTD+CR+HR1+CH+CD | CDP5T-S2 | 57 aa | Chain1\| 712, 713, 714, 715, 907, 909, 910, 912, 913, 1037, 1038, 1039, 1040, 1041, 1042, 1043, 1044, 1045, 1046, 1047, 1048, 1066, 1067, 1071, 1072, 1091, 1092, 1093, 1094, 1095, 1105, 1106, 1107, 1108, 1109. Chain2\| 884, 885, 886, 887, 889, 890, 891, 892, 894, 895, 896, 900, 901, 904, 905, 907, 913, 1037, 1038, 1039, 1040, 1041 |
|  | S2 | NTD+CH | CDP6T-S2 | 15 aa | Chain1\| 725, 1024, 1025, 1028, 1029, 1039, 1040, 1041, 1042. Chain2\| 780, 784, 1026, 1027, 1030, 1031 |
|  | S2 | NTD+CH | CDP7T-S2 | 11 aa | 738, 739, 742, 753, 754, 756, 757, 758, 759, 760, 761, 1001 |
|  | S2 | NTD+FP+CR+HR1+CH+CD | CDP8T-S2 | 19 aa | Chain1\| 707, 709, 710, 711, 1077, 1078, 1079, 1129, 1130, 1131 Chain2\| 796, 797, 798, 897, 898, 899, 900, 917, 920. |
|  | S2 | CR+HR1+CH+CD | CDP9T-S2 | 18 aa | Chain1\| 900, 903, 904, 907, 913, 917. Chain2\| 1077, 1078, 1079, 1080, 1089, 1090, 1091, 1092, 1093, 1094, 1095, 1107 |
|  | S2 | CR+HR1+CH+CD | CDP10T-S2 | 17 aa | 910, 912, 914, 1091, 1092, 1093, 1104, 1105, 1106, 1107, 1108, 1109, 1113, 1118, 1119, 1121, 1122 |
|  | S2 | CD | CDP11T-S2 | 10 aa | 1101, 1102, 1103, 1113, 1114, 1115, 1135, 1136, 1137, 1138 |
|  | S1+S2 | S1-NTD +S2-NTD + CR + HR1 + CH | CDP1T-S1S2 | 40 aa | 42, 43, 44, 45, 46, 47, 48, 49, 737, 739, 740, 741, 742, 743, 744, 745, 746, 750, 853, 854, 855, 856, 857, 858, 963, 964, 966, 967, 975, 976, 977, 978, 979, 982, 983, 993, 996, 997, 998, 1000 |
|  | S1+S2 | S1-NTD + S2-NTD + CR + HR1 + CH | CDP2T-S1S2 | 71 aa | Chain1\| 42, 43, 44, 45, 46, 47, 48, 49, 737, 739, 740, 741, 742, 743, 744, 745, 746, 750, 853, 854, 855, 856, 857, 858, 963, 964, 966, 967, 975, 976, 977, 978, 979, 982, 983, 993, 996, 997, 998, 1000. Chain2\| 319, 320, 321, 322, 536, 537, 538, 541, 542, 546, 547, 548, 549, 550, 565, 567, 568, 569, 570, 571, 572, 573, 574, 576, 587, 588, 589, 590, 591, 592, 619 |
|  | S1+S2 | S1-NTD+ RBD + SD1 + S2-HR1 | CDP3T-S1S2 | 63 aa | 328, 329, 330, 331, 332, 333, 360, 361, 362, 365, 366, 368, 369, 370, 377, 379, 382, 384, 385, 387, 388, 390, 391, 392, 393, 431, 432, 434, 513, 515, 516, 517, 518, 519, 520, 521, 522, 523, 524, 525, 526, 527, 528, 529, 530, 543, 544, 545, 546, 547, 564, 565, 567, 573, 576, 577, 578, 579, 580, 582, 585, 982, 983 |
|  | S1+S2 | S1-RBD + S2-CH | CDP4T-S1S2 | 14 aa | 368, 369, 370, 371, 372, 374, 377, 378, 379, 380, 381, 384, 985, 987 |
|  | S1+S2 | S1-NTD + SD2 + S2-NTD + CR | CDP5T-S1S2 | 42 aa | Chain1\| 302, 312, 313, 314, 315, 316, 318, 592, 593, 594, 596, 611, 613, 614, 646, 647, 665, 666, 667. Chain2\| 733, 735, 736, 737, 738, 740, 761, 763, 764, 765, 766, 767, 768, 771, 772, 775, 857, 858, 859, 861, 862, 863, 864. |
|  | S1+S2 | S1-SD2; S2-NTD + CR | SP1T-S1S2 | 9 aa | 661, 662, 697, 698, 699, 700, 783, 865, 873 |
