# Supplementary material for: Unlocking COVID therapeutic targets: A structure-based rationale against SARS-CoV-2, SARS-CoV and MERS-CoV Spike
Source: Comput Struct Biotechnol J. 2020 Jul 31;18:2117–31. doi: 10.1016/j.csbj.2020.07.017 (PMC7452956; doi:10.1016/j.csbj.2020.07.017)
Supplement: Supplementary table 3 — Top-ranked hot spots for drug targeting among beta-CoVs. The T-RHS for each group (1) S monomer (2) S-RBD and (3) S trimer were predicted based on the descriptors algorithm from all the pocket bioinformatics tools: SF, DGSS and PDS, with respected to the hSARSr-CoVs and the SARSr- and MERSr-CoVs. [file mmc6.docx]

**Table S-3. Top-ranked hot spots for drug targeting among beta-CoVs**. The T-RHS for each group (1) S monomer (2) S-RBD and (3) S trimer were predicted based on the descriptors algorithm from all the pocket bioinformatics tools: SF, DGSS and PDS, with respected to the hSARSr-CoVs and the SARSr- and MERSr-CoVs.

|  | **human SARSr-CoV** | | | **SARSr- and MERSr-CoV** | | |
| --- | --- | --- | --- | --- | --- | --- |
|  | **S1** | **S2** | **Total Number**  **(S1 + S2)** | **S1** | **S2** | **Total Number**  **(S1 + S2)** |
| **Spike Monomer** | Y38, D40, F43, F58, V83, R102, W104, N121, I128, C136, P139, F140, L189, R190, F192, P225, L242, G283, D287, A288, V289, D290, L296, K300, R319, V320, C336, F338, G339, F342, N343, A344, F347, V362, A363, D364, V367, L368, F374, Y380, K386, F392, V407, Q409, I410, A411, I418, P426, F429, V433, W436, D442, R509, S514, F515, G526, P527, K528, C538, L546, T549, P589, C590, S591, F592 | V729, S730, M740, Y741, I742, C743, G744, T778, F782, Y789, K790, T791, K795, F797, S803, Q804, L806, P807, D808, P809, R815, S816, F817, F855, N856, P863, L865, T866, D867, I870, A871, T883, Q895, I896, P897, F898, G908, I909, G910, T912, Q954, A958, T961, L962, Q965, L966, S975, V976, L977, R1000, S1003, Y1007, Q1010, R1014, Q1036, S1037, K1038, R1039, V1040, D1041, G1046, Y1047, H1048, A1056, P1057, H1058, R1091, E1092, G1093, W1102, T1105, Q1106, I1115, N1135, T1136, Y1138 | (65+76) = 41 | Y38, F58, V83, N121, F140, L189, R190, F192, G283, A288, D290, L296, C336, F338, L368, F392, I418, F515, K528, C538, C590. | Y741, C743, F782, F797, P807, R815, S816, P863, A871, I896, P897, F898, G908, G910, T912, A958, L962, L966, R1000, Y1007, Q1010, R1014, Q1036, S1037, K1038, R1039, G1046, H1048, A1056, P1057, G1093, T1105, I1115. | (21+33) = 54 |
|  | **human SARSr-CoV** | | | **SARSr- and MERSr-CoV** | | |
|  | **S1** | **S2** | **Total Number**  **(S1 + S2)** | **S1** | **S2** | **Total Number**  **(S1 + S2)** |
| **Spike-RBD** | F338, G339, F342, N343, R355, Y365, S366, V367, L368, Y369, S371, F374, T376, F377, K378, C379, Y380, T385, L387, N388, Y396, V407, R408, I410, A411, P426, D428, F429, C432, V433, W436, R454, R457, F464, D467, S469, Y473, P491, L513, S514, F515, E516, C525 |  | 43 | F338, R355, L368, F377, C379, C432, D467, F515, C525 |  | 9 |
|  | **human SARSr-CoV** | | | **SARSr- and MERSr-CoV** | | |
|  | **S1** | **S2** | **Total Number**  **(S1 + S2)** | **S1** | **S2** | **Total Number**  **(S1 + S2)** |
| **Spike trimer** | P39, D40, F43, R44, S45, L48, F58, F86, I128, C136, P139, F140, T167, F168, L189, K195, N196, D198, Y200, Y204, P225, L229, P230, G232, I233, N234, I235, L242, N282, D287, V289, D290, L296, Q314, P322, F338, G339, N343, F347, W353, Y365, Y369, T376, F377, C379, L387, L390, Y396, V407, A411, G413, A419, K424, D427, F429, R454, P463, F464, R466, R509, S514, E516, L517, L518, V524, C525, G526, P527, F541, N544, L546, T547, G548, T549, G550, K557, F562, R567, D568, D571, T573, D574, R577, I587, P589, C590, S591, F592, G593, G594, S596, L611, Q613, A647, E661, C662, P665, I666, G667 | M697, S698, L699, G700, N709, N710, I712, A713, I714, P715, E725, P728, V729, S730, K733, S735, V736, D737, C738, M740, Y741, I742, C743, G744, D745, S746, L753, L754, Y756, G757, S758, F759, C760, T761, Q762, L763, N764, R765, A766, G769, I770, A771, E773, D775, N777, T778, E780, F782, Y789, K790, T791, K795, G798, L806, P807, R815, K854, F855, N856, G857, T859, V860, L861, P862, P863, L864, D867, I870, A871, Y873, G885, W886, T887, G889, A890, G891, L894, Q895, I896, P897, M900, Q901, Y904, N907, G908, G910, T912, Q913, N914, Y917, Q920, K947, D950, V951, Q954, Q957, A958, T961, L962, K964, Q965, L966, S967, F970, S975, V976, L977, N978, D979, S982, R983, D985, V987, D994, R995, T998, G999, L1001, Q1002, S1003, Q1005, T1006, Y1007, T1009, Q1010, L1012, I1013, R1014, A1015, A1016, E1017, I1018, R1019, S1021, L1024, A1026, T1027, K1028, S1030, E1031, G1035, Q1036, S1037, K1038, R1039, V1040, D1041, F1042, C1043, G1044, K1045, G1046, Y1047, H1048, A1056, P1057, H1058, G1059, T1077, A1078, P1079, P1090, R1091, E1092, G1093, V1094, T1105, Q1106, R1107, N1108, Q1113, I1114, I1130, G1131 | (99+174) = 273 | P39, S45, F58, F86, F140, L189, L229, P230, I235, D290, L296, P322, F338, F377, C379, K424, V524, C525, F541, G548, G550, D568, C590, S596, L611, C662, P665, G667. | I714, P715, E725, K733, V736, D737, C738, Y741, C743, L753, Y756, G757, F759, C760, N764, A766, G769, D775, F782, G798, P807, R815, G857, V860, L861, P862, P863, L864, A871, Y873, G885, W886, T887, G889, I896, P897, Y904, N907, G908, G910, T912, Q913, Q920, K947, D950, Q957, A958, L962, L966, S967, F970, D979, R983, D985, D994, R995, G999, L1001, Y1007, Q1010, L1012, R1014, S1021, L1024, K1028, E1031, Q1036, S1037, K1038, R1039, F1042, C1043, G1044, G1046, H1048, A1056, P1057, G1059, A1078, P1090, G1093, T1105. | (28+82) = 110 |
