# Supplementary material for: Unlocking COVID therapeutic targets: A structure-based rationale against SARS-CoV-2, SARS-CoV and MERS-CoV Spike
Source: Comput Struct Biotechnol J. 2020 Jul 31;18:2117–31. doi: 10.1016/j.csbj.2020.07.017 (PMC7452956; doi:10.1016/j.csbj.2020.07.017)
Supplement: Supplementary table 6 — Conserved druggable sites/residues for drug targeting shared by the Spike monomer and trimer structures. The CDR shared by both groups (S monomer and trimer) were predicted based on the descriptors algorithm of the pocket bioinformatics tools: SF and DGSS, with respected to the hSARSr-CoVs and the SARSr- and MERSr-CoVs. [file mmc9.docx]

**Table S-6. Conserved druggable residues for drug targeting shared by the Spike monomer and trimer structures.** The CDR shared by both groups (S monomer and trimer) were predicted based on the descriptors algorithm of the pocket bioinformatics tools: SF and DGSS, with respected to the hSARSr-CoVs and the SARSr- and MERSr-CoVs.

|  | **human SARSr-CoV** | | | **SARSr- and MERSr-CoV** | | |
| --- | --- | --- | --- | --- | --- | --- |
| **Spike: Monomer + Trimer** | **S1** | **S2** | **Total Number**  **(S1 + S2)** | **S1** | **S2** | **Total Number**  **(S1 + S2)** |
|  | R34, Y38, D40, V42, F43, R44, H49, F58, F59, F65, I81, P82, V83, F86, D88, F92, T95, N99, I100, I101, R102, G103, W104, I105, T109, L110, S116, I119, V120, N121, V126, I128, F133, C136, P139, F140, L189, R190, F192, F194, N196, I203, H207, I210, P217, G219, F220, I233, N234, I235, L242, A263, Y266, V267, T284, D287, A288, V289, D290, L296, E298, K300, L304, F306, T315, S316, R319, V320, P322, F329, P330, N331, I332, L335, C336, P337, F338, G339, E340, F342, N343, A344, T345, R346, F347, W353, R355, K356, C361, V362, A363, D364, Y365, S366, V367, L368, Y369, F374, T376, F377, K378, Y380, T385, K386, L387, N388, L390, C391, F392, N394, V395, Y396, S399, V407, R408, Q409, I410, A411, P412, G413, Q414, I418, A419, Y421, N422, Y423, K424, L425, P426, D427, D428, F429, C432, V433, W436, D442, N448, Y451, F464, R466, R509, L513, S514, F515, L517, V524, C525, G526, P527, K528, S530, C538, F541, N544, G545, L546, G548, T549, G550, V551, F565, T573, R577, I587, P589, C590, S591, F592, V615, E619, D663 | Y707, I712, A713, P728, V729, S730, M731, K733, T734, M740, Y741, I742, C743, G744, I770, Q774, D775, T778, F782, Y789, K790, T791, K795, F797, S803, Q804, L806, P807, P809, Q814, R815, S816, F823, F855, N856, V860, L861, P863, L864, L865, T866, D867, E868, I870, A871, G885, W886, T887, L894, Q895, I896, P897, F898, Q901, Y904, G908, I909, G910, T912, K947, D950, V951, V952, Q954, N955, A956, Q957, A958, L959, N960, T961, L962, Q965, L966, S975, V976, L977, R1000, S1003, T1006, Y1007, Q1010, R1014, A1015, Q1036, S1037, Q1038, R1039, V1040, D1041, F1042, C1043, G1044, K1045, G1046, Y1047, H1048, A1056, P1057, H1058, G1059, P1090, R1091, E1092, G1093, W1102, F1103, V1104, T1105, Q1106, R1107, N1108, Q1113, I1114, I1115, N1119, N1135, T1136, Y1138 | (171+119) = **290** | Y38, F58, F86, D88, T95, N99, I100, I101, G103, I105, T109, S116, N121, V126, P139, F140, L189, R190, F192, F194, I203, I210, G219, N234, I235, A263, Y266, V267, A288, D290, L296, S297, E298, L304, F306, S316, V320, P322, P330, N331, C336, F338, F342, N343, A344, T345, R355, C361, D364, L368, S373, F377, P384, T385, L387, C391, F392, N394, V395, Q409, I410, A411, G413, I418, A419, Y421, N422, Y423, K424, P426, C432, V433, I434, N437, Y451, L513, S514, F515, L517, V524, C525, P527, K528, S530, C538, F541, G545, L546, G548, G550, V551, F565, P589, C590, V615 | M731, Y741, I742, C743, Q774, D775, F782, F797, P807, R815, S816, F823, V860, L861, P863, A871, G885, W886, T887, I896, P897, F898, Y904, G908, I909, G910, T912, K947, D950, V952, N955, A956, Q957, A958, L959, L962, L966, R1000, Y1007, Q1010, R1014, Q1036, S1037, Q1038, R1039, F1042, C1043, G1044, G1046, H1048, A1056, P1057, G1059, P1090, G1093, W1102, T1105, I1115, N1119 | (95+59) = **154** |
