# Supplementary material for: Unlocking COVID therapeutic targets: A structure-based rationale against SARS-CoV-2, SARS-CoV and MERS-CoV Spike
Source: Comput Struct Biotechnol J. 2020 Jul 31;18:2117–31. doi: 10.1016/j.csbj.2020.07.017 (PMC7452956; doi:10.1016/j.csbj.2020.07.017)
Supplement: Supplementary data 11 [file mmc11.docx]

**Supplementary information – Results and Discussion**

### Spike monomer conformation

In regard to the S monomer structure, 72 potential druggable pockets (out of 156) were identified (DGSS server) in both open (39 pockets) and closed (33 pockets) state conformations. Several pockets were found to be shared by all the structures, independently of the RBD conformational switches. The supplemental **Table S-1** displays the pockets identified for each S monomer structure, along with the pocket descriptors: size, volume and druggability score.

A linear schematic representation of the S monomer (in closed and open conformations) containing druggability information for each aa position is shown in the **Figure S-2**. Some differences are revealed in the distribution of druggable aa regions/residues through the S monomer, along with some variations between the two conformations. These may indicate distinct modes of interaction between each S domain and host proteins or other molecules. For the comparative analysis between the S monomer conformations, only residues or regions graded with druggability score ≥3 for one conformation while <3 for the opposite conformation were considered, according to the monomer druggability score scale (0-4) depicted in the Figure S-2". Overall, in S1, a higher number of additional sites/residues were found for the closed conformation. This was observed in the S1-NTD, RBD and SD1, but not in the SD2 where additional druggable residues were only found for the open conformation. In the S2 subunit, a distinct pattern was observed, with a higher number of additional residues found for the open conformation, except in the CR, CH and CD. The additional residues, found either for the open or closed conformation, do not directly imply a significant higher number of potential pockets identified for a specific S monomer conformation.

Conserved druggable regions common to both S open and closed states have been identified. These are located close together forming larger pockets. In this context, 23 main consensus pockets, named consensus druggable pockets (CDP), (18 CDPs with ≥ 14 aa) were found in the S monomer (CDPM), 14 were identified in S1 (CDPM-S1) and 9 in S2 (CDPM-S2). The supplemental **Table S-2** displays the CDPs identified for the S monomer conformation (along with the aa number and composition; and the corresponding location/domain within the protein structure.

The CDPs were sorted according to the protein sequence location (from the N-terminal to the C-terminal). In S1, the first 6 CDPs (CDP1M-S1 to CDP6-S1) are located in the S-NTD. The following 6 CDPs (CDP7M-S1 to CDP12M-S1) are located in the S1-RBD. The CDP13M-S1 is located in the S1-SD1 and the CDP14M-S1 is mainly located in the SD1 and SD2. In S2 ( CDPs with ≥ 14 aa), the CDP1M-S2 is located at the NTD, FP, HR1 and CH regions; CDP3M-S2 in the NTD, HR1 and CH regions,; CDP4M-S2 in the FP and CH regions; CDP5M-S2 in the FP; CDP6M-S2 in the CR and CH; CDP7M-S2 in the HR1 and CD; and CDP8M-S2 and CDP9M-S2 in the CH. In the S open conformation, CDP8M-S1-to-CDP12M-S1 can assemble in two distinct conformations to form two larger pockets within the S1-RBD. Moreover, for the 6VXX structure (down RBD conformation) is possible to identify a larger 65-aa long pocket in the S2 subunit (including the FP, HR1 and CH domains), comprising pockets CDP1M-S2 and CDP4M-S2.

Distinct RBD conformations do not have a significant impact on the number of potential pockets identified for each S monomeric state. However, they can determine the rearrange of pockets (that can merge together) common to both conformations, since it is possible to predict larger pockets either in the S1-RBD subunit for the open conformation or in the S2-subunit for the closed conformation.

In the final analysis of the monomer conformation, a comparative analysis of the druggability consensus and the conservation score was performed for the hSARSr-CoVs and for the SARSr- and MERSr-CoVs (supplemental **Figure S-3**). The conserved druggable regions are marked with an asterisk and the T-RHS are marked with a target.

The 141 T-RHS for drug targeting (out of 313 CDR) identified in hSARSr-CoVs comprise 65 hot spots in S1 (out of 182 CDR) and 76 hot spots in S2 (out of 131 CDR). The **Table S-3** displays the CDR and T-RHS identified for the S monomer conformation. No major differences were found between the subunits with respect to the major prevalence of CDR, with 27% (182/685) in S1 and 22% (131/588) in S2; or to the prevalence of T-RHS, with 9.5% (65/685) in S1 and 12.9% (76/588) in S2. It has been found that 47.4% of the CDR in S1 are located in the RBD (81 out of 182 CDR) and that the RBD is the most druggable domain within S1 (81 conserved druggable hot spots out of the 195 overall domain residues). SD2 appears to be less relevant (5 druggable residues out of a total of 94 aa) in regard to the druggability estimates. For the S2 subunit, most of the CDR are located in the regions which comprise FP, HR1, CR and CH. The FP was found to be the most druggable domain (9 druggable residues out of a total 19 aa).

The 54 T-RHS for drug targeting (out of 164 CDR) found in the SARSr- and MERSr-CoVs comprise 21 hot spots (out of 103 CDR) in the S1 subunit and 33 hot spots (out of 61 CDR) in the S2 subunit (**Table S-3**). The majority of the S1 CDR are located in the RBD (45 out of 103 conserved druggable hot spots). The S1-RBD is also the most conserved druggable domain. For the S2 subunit, most of the CDR are also located in the regions that comprise the FP, CR, HR1 and CH. The majority of the T-RHS are located in the CH.

No major differences were found when comparing the absolute number of CDR between the S1 and S2 subunits (<10%). Albeit, it is interesting to notice that a slightly higher number of T-RHS were found in S2 and not in S1 where the RBD is located, suggesting that other regions than S1-RBD bear great druggable potential and should be further addressed in anti-CoV drug discovery strategies. Taking all together, the most promising conserved regions regarding the druggability potential of the monomer conformation are the S1-RBD and the FP, CR, HR1 and CH in the S2 subunit.
